# Efferocytosis-associated genes serve as prognostic biomarkers for pancreatic ductal adenocarcinoma and identify P2RY6 as a therapeutic target

**DOI:** 10.3389/fimmu.2025.1708441

**Published:** 2025-11-26

**Authors:** Xiangjun Wang, Wei Wang, Chuanxin Yang, Puxiongzhi Wang, Yangming Liu, Liqin Yu, Sijie Zhang, Xiaoyu Yan, Jian Wang

**Affiliations:** Department of Hepatobiliary and Pancreatic Surgery, Shanghai Sixth People’s Hospital Affiliated to Shanghai Jiao Tong University School of Medicine, Shanghai, China

**Keywords:** pancreatic ductal adenocarcinoma, efferocytosis, prognostic signature, immunosuppressive microenvironment, P2RY6, MRS-2578

## Abstract

**Background:**

Pancreatic ductal adenocarcinoma (PDAC) is a highly malignant tumor with poor prognosis. Efferocytosis, an essential process for clearing apoptotic cells, is involved in shaping immunosuppressive microenvironment, facilitating tumor immune evasion. This study aims to evaluate the prognostic value of efferocytosis-related biomarkers in PDAC and elucidate their underlying mechanisms, providing insights for personalized therapy.

**Methods:**

We integrated bulk and single-cell transcriptomic data from public database to construct and validate an efferocytosis-related prognostic model for PDAC. Additionally, we analyzed the specimens from a cohort of 81 PDAC patients alongside cell experiments to elucidate the function of P2RY6. RNA-seq analysis was employed to uncover the effector pathways mediated by P2RY6.

**Results:**

An efferocytosis-based prognostic model, EFFscore, developed based on *ADAM9*, *P2RY6*, and *CD36*, can effectively assess the tumor phenotype of PDAC patients. The EFFscore is strongly associated with tumor evolution, malignant biological characteristics and microenvironmental interactions of PDAC. The essential mediator P2RY6 is significantly upregulated in PDAC tissue and cells, correlating closely with poor prognosis. Functional studies demonstrate that P2RY6 inhibition exhibits tumor-suppressive effects by activating the endoplasmic reticulum stress and enhancing anti-tumor immune responses. The P2RY6 receptor inhibitor, MRS-2578, emerges as a promising therapeutic candidate for PDAC treatment.

**Conclusion:**

The prognostic model EFFscore exhibits remarkable predictive performance, accurately reflecting the malignant potential of PDAC. P2RY6 serves as a key oncogenic factor driver in PDAC, its targeted inhibition significantly suppresses tumor progression, highlighting its dual potential as a diagnostic biomarker and therapeutic target.

## 1 Introduction

Pancreatic ductal adenocarcinoma (PDAC), the most common pathological subtype of pancreatic cancer, is characterized by an extremely high mortality rate, with a five-year overall survival (OS) rate of only 13% (1). PDAC originates from the exocrine tissue of the pancreas. Malignant transformation of acinar and ductal cells is driven by oncogenic *KRAS* mutations and the functional loss of tumor suppressor genes such as *TP53* and *CDKN2A* (2). This neoplastic transformation typically proceeds through precancerous lesions, including pancreatic intraepithelial neoplasia (PanIN) and intraductal papillary mucinous neoplasm (IPMN), eventually evolving into invasive carcinoma (2). Despite its prolonged development, its insidious onset and rapid progression of PDAC often delay diagnosis until advanced stages, greatly limiting treatment efficacy and long-term survival (3). Current standard therapies for PDAC include surgical resection, chemotherapy, and, more recently, targeted and immunotherapies. However, PDAC generally exhibits poor responsiveness to chemotherapy and immunotherapy, resulting in limited clinical benefits for patients with advanced disease (4). Moreover, conventional tumor biomarkers (e.g., CA125, CA19-9) and emerging candidates (e.g., microRNAs and circulating tumor DNA) have not achieved the sensitivity and specificity required for precision medicine (4, 5). Therefore, enhancing early detection rates and identifying novel biomarkers and therapeutic targets remain urgent priorities for improving PDAC patient outcomes.

In recent years, immunotherapy has achieved remarkable success across multiple cancer types. However, PDAC remains largely unresponsive, except for the rare (<1%) microsatellite instability–high (MSI-H) subtype (6). This resistance is primarily attributed to PDAC’s characteristic driver mutations, highly immunosuppressive tumor microenvironment (TME), and dense stromal barrier (6). Mutant KRAS (mKRAS), present in over 90% of PDAC cases, not only drives oncogenic signaling but also fosters immunosuppression by downregulating cell surface MHC-I and upregulating CD47 and PD-L1 (7, 8). Furthermore, the TME—enriched with immunosuppressive cell populations and compounded by poor vascularization—severely limits immune infiltration and effector function (2). Overcoming these immunosuppressive barriers and identifying novel immune-related therapeutic targets are therefore critical for improving PDAC patient outcomes.

Efferocytosis, the process by which phagocytes clear apoptotic cells has emerged as a critical contributor to the immunosuppressive microenvironment of solid tumors, particularly PDAC (9). The dense fibrotic extracellular matrix (ECM) in PDAC creates a hypoxic and nutrient-deprived microenvironment that imposes selective pressure, inducing apoptosis (2). Efferocytosis efficiently eliminates apoptotic cells by recognizing “eat-me” signals like phosphatidylserine (PS) through receptors including Brain-specific angiogenesis inhibitor 1 (BAI1) and MER proto-oncogene, tyrosine kinase (MerTK). This process prevents secondary necrosis and inflammation, thus supporting tumor cell survival and immune evasion (10). Following efferocytosis, tumor-associated macrophages (TAMs) undergo metabolic reprogramming mediated by Peroxisome proliferator-activated receptor gamma (PPARγ) and Liver X receptor (LXR), leading to their polarization towards an M2-like phenotype. These immunosuppressive TAMs secrete anti-inflammatory cytokines such as Interleukin-10 (IL-10) and Transforming growth factor-β (TGF-β), which inhibit effector T cell function and facilitate immune escape (11–14). Moreover, efferocytosis impairs dendritic cell maturation and antigen presentation, inhibits T cell proliferation, remodels cytokine profiles toward immunosuppression, and induces immune checkpoint expression such as PD-L1 on tumor cells (15). Despite increasing insights into efferocytosis-driven immunosuppression, its clinical implications in PDAC, characterized by a profoundly immunosuppressive microenvironment, remain poorly understood. Therefore, identifying and characterizing efferocytosis-related biomarkers is essential to improve prognosis prediction and inform novel therapeutic strategies in PDAC.

Extracellular purines and pyrimidines are important signaling molecules released by most tissues and organs. They can regulate cellular functions through the activation of purinergic receptors (16). The P2RY6 receptor, a G-protein-coupled receptor (GPCR) primarily activated by extracellular UDP regulates cell proliferation, migration, and immune responses (17). Previous studies have demonstrated that P2RY6 has significant pro-inflammatory effects in various immune cells. Its blockade attenuates the progression of inflammatory diseases such as inflammatory bowel disease (IBD), acute lung injury (ALI), and atherosclerosis (18, 19). However, P2RY6 contributes to immunosuppression rather than inflammation within the TME. For example, UDP released by PDAC cells can engage P2RY6 receptor to recruit TAMs, promoting immunosuppression (20). In addition, recent studies indicate that tumor-intrinsic P2RY6 also exerts oncogenic effects and correlates with poor prognosis (21). For instance, endogenous P2RY6 in breast cancer cells promotes the synthesis of prostaglandin E2 (PGE2) through activation of the Gq/Phospholipase C beta (PLC-β) pathway, thereby driving immune suppression (22). Although P2RY6 has been implicated in tumor progression across various cancers, its endogenous function and underlying mechanisms in PDAC remain poorly defined.

This study identifies significant enrichment of the efferocytosis-related pathways in PDAC tissue. Based on the efferocytosis pathway gene set, we developed a PDAC prognostic model, EFFscore, which demonstrates remarkable predictive capability in terms of patient prognosis, genome variation, immune infiltration, and drug resistance. Additionally, scRNA-seq analysis revealed the intrinsic association between EFFscore and the tumor evolution, malignant biological characteristics and microenvironmental interactions of PDAC. Among the efferocytosis-associated genes, *P2RY6* emerged as a particularly compelling candidate characterized by its distinct expression profile and significant correlation with poor clinical outcomes. Functional assays confirmed that P2RY6 is significantly upregulated in both PDAC tissues and cell lines, and its inhibition markedly attenuates the malignant phenotype of PDAC cell both *in vitro* and *in vivo*. Transcriptomic analysis indicates that P2RY6 knockdown exerts tumor-suppressive effects through excessively activating the endoplasmic reticulum stress (ERS) and enhancing anti-tumor immune responses. In conclusion, this study constructs a novel three-gene efferocytosis-related prognostic model and highlights the critical role of P2RY6 in PDAC progression, providing new insights for clinical diagnosis and therapy in PDAC.

## 2 Methods

### 2.1 Patients and specimens

The samples in this study were obtained from patients between May 1, 2016, and May 31, 2018. A total of 81 patients diagnosed with primary PDAC were included, and corresponding adjacent normal tissue were collected from 44 of these patients as controls. All clinical pathological data were extracted from the patients’ medical records. Informed consent was obtained from all participants, and the study was approved by the institutional ethics committee.

### 2.2 Antibodies and reagents

The following antibodies and reagents were used in this study:anti-P2RY6 (107142-T44, SinoBiological, China), anti-β-Actin (66009-1-IG, Proteintech, China), anti-ADAM9 (CSB-PA618774ESR1HU, CUSABIO, China), anti-CD36 (WL02390, Wanleibio, China), anti-DDIT3 (Abmart, T56694, China), anti-HSPA5 (SinoBiological, 102056-T44, China), anti-PERK (A18196, ABclonal, China), anti-BAX (50599-2-IG, Proteintech, China), anti-BCL2 (12789-1-AP, Proteintech, China), anti-ERO1A (CSB-PA846631EDR1HU,CUSABIO, China), anti-TNFRSF10B (WL0171, Wanleibio, China), anti-PPP1R15A (WL05554, Wanleibio, China). MRS-2578 (HY-13104, MedChemExpress, USA), 4-PBA (HY-A0281, MedChemExpress, USA). The sequences of primers used in this study are listed in **Supplementary Table S1**.

### 2.3 Data extraction and patient information preprocessing

Transcriptomic data and corresponding clinical characteristics of pancreatic adenocarcinoma (PAAD) were obtained from The Cancer Genome Atlas (TCGA) database (https://portal.gdc.cancer.gov) and used as the experimental cohort. Clear inclusion and exclusion criteria were applied: only primary tumor samples were retained, while patients with incomplete transcriptomic profiles or missing essential clinical information (e.g., survival time or status) were excluded. A total of 178 patient samples were included after filtering, ensuring data consistency and reliability for downstream analyses. Normal pancreatic tissue transcriptomic data (n=167) were retrieved from the Genotype-Tissue Expression Project (GTEx) database (https://www.gtexportal.org/) as the control cohort. To minimize batch effects, data normalization was performed using the R “limma” package. Additionally, to ensure the reliability of the results, gene expression matrices and corresponding clinical and survival data from the GEO database were downloaded for validation, specifically from GSE62452 (n = 65) and GSE183795 (n = 134) (https://www.ncbi.nlm.nih.gov/geo/) (23, 24).

### 2.4 Construction and validation of prognostic signature model based on efferocytosis-related genes

Differentially expressed genes (DEGs) were identified in PDAC samples from TCGA and normal control samples from GTEx, using |log2FC| > 1 and adjusted p < 0.05 as thresholds, resulting in 5,871 DEGs. These DEGs were then intersected with 156 genes from the efferocytosis pathway in Kyoto Encyclopedia of Genes and Genomes (KEGG), yielding 68 differentially expressed efferocytosis-related genes (DE-ERGs). Gene Ontology (GO) and functional annotation analyses of these genes were conducted using the R “ClusterProfiler” package, with a significance threshold set at p < 0.05. In the TCGA training cohort, univariate Cox regression, LASSO regression, and multivariate Cox regression analyses were performed to construct the prognostic model, EFFscore. Patients were divided into high and low EFFscore groups based on the median score. The predictive performance and reliability of the model were systematically evaluated using receiver operating characteristic (ROC) curves, Kaplan-Meier (K-M) survival curves, and survival status and risk score distribution plots.

### 2.5 Analysis of clinical transcriptomic features

Construction of Calibration Curve and Nomogram: A nomogram was developed using the R “RMS” package, integrating EFFscore with clinical characteristics to assess individual survival probabilities (25). The calibration curve was used to validate the consistency between predicted and actual survival data, while ROC curves were employed to evaluate the predictive accuracy of the nomogram. Analysis of Somatic Mutations and Copy Number Variation (CNV): Somatic mutation data were sourced from the TCGA database. Tumor mutation burden (TMB) was defined as the total number of somatic coding errors, base substitutions, and insertion-deletion mutations per megabase. Somatic mutation data were visualized using the R “Maftools” package, with waterfall plots displaying common mutated genes and their distribution patterns in PDAC samples (26). The GRCh38 reference genome was used for annotating genes in CNV regions. Immune Infiltration and Prediction of Immunotherapy Response: The ESTIMATE algorithm was used to calculate immune scores, ESTIMATE composite scores, and tumor purity (27). The relative abundance of immune infiltration was evaluated using the R “CIBERSORT” and “GSVA” packages, employing both CIBERSORT and ssGSEA algorithms. Additionally, the TIDE (Tumor Immune Dysfunction and Exclusion) algorithm was used to predict potential responses of PDAC patients to immune checkpoint inhibitor therapy (28). Prediction of Chemotherapy Drug Sensitivity: The R “OncoPredict” package was utilized to assess the sensitivity of each sample to a variety of drugs by calculating the half maximal inhibitory concentration (IC50) for common targeted and chemotherapeutic agents (29). IC50 data were sourced from the Cancer Therapeutics Response Portal (CTRP, https://portals.broadinstitute.org/ctrp/).

### 2.6 Processing of scRNA-seq data

scRNA-seq data (GSE194247; GSE235449; GSA: CRA001160) were processed using the R “Seurat” package (v5.0.1) (30, 31). The filtering criteria were as follows: cells with fewer than 500 genes, a mitochondrial gene proportion greater than 15%, or a gene count per cell not falling between 500 and 5000 (32). The data were normalized using the “NormalizeData” function (LogNormalize), and the top 2000 highly variable genes were selected for principal component analysis (PCA). Uniform Manifold Approximation and Projection (UMAP) was applied for data visualization. Doublets were identified using the R “DoubletFinder” package (v2.0.3) with a 5% doublet rate assumption, and sample integration was performed using the R “Harmony” package (33, 34). Cluster-specific genes were identified and matched with existing literature and the CellMarker database (http://117.50.127.228/CellMarker/) to classify the cell types within each cluster.

### 2.7 Analysis of scRNA-seq data

Inference of CNV: R “inferCNV” package (v1.6.0) was utilized to infer large-scale chromosomal CNV level in somatic cells (35). The analysis employed the gene expression matrix of ductal cells, annotation data, and gene/chromosome location information. Non-ductal cells were designated as reference cells without CNV. CNV scores for each cell cluster were determined by calculating the second derivative of CNV regions. Pseudotime Trajectory Analysis: pseudotime and trajectory analysis was conducted using R “Monocle3” package (v1.3.7) (36). Potential discontinuous trajectories were constructed via the graph learning function, and pseudotime was defined using the “order_cells” function, with a specific node as the starting point. This approach simulated the dynamic evolutionary progression of PDAC cells. Differentially expressed genes along the pseudotime trajectory were identified, and pseudotime scores and trajectory distributions were visualized on UMAP plots. The expression trends of these genes along the pseudotime trajectory were illustrated using the “plot_genes_in_pseudotime” function. Gene Set Variation Analysis (GSVA): Pathway activation differences between groups were assessed using the GSVA method, implemented via R “clusterProfiler package.” Pathway data were obtained from the Molecular Signatures Database (MSigDB, https://www.gsea-msigdb.org/gsea/msigdb) (37). Cell-Cell Interaction Network Analysis: Intercellular communication events were analyzed using the R “CellChat” package (v1.6.1) (38). Based on the molecular characteristics of ligand-receptor pairs, communication events were primarily defined as secreted signaling. Interactions between different cell types were visualized using connecting lines to represent communication events.

### 2.8 Cell culture

The human PDAC cell lines used in the experiments included CAPAN-1, MIA-PaCa2, PANC-1, BxPC-3 and AsPC-1, alongside the normal pancreatic ductal epithelial cell line hTERT-HPNE, all obtained from the American Type Culture Collection (ATCC, Manassas, VA). Cell culture media were supplemented with 10% fetal bovine serum (FBS) and 1% penicillin/streptomycin. DMEM/F12 medium (GIBCO) was used for HPNE, MIA-PaCa2, and PANC-1, while RPMI 1640 medium (GIBCO) was used for BxPC-3 and AsPC-1. CAPAN-1 was cultured in IMDM medium (GIBCO). All cell lines were routinely tested to confirm the absence of mycoplasma contamination, ensuring the reliability of experimental outcomes.

### 2.9 RNA extraction and analysis of gene and protein expression

Total RNA was extracted from cells and tissues using Trizol reagent (15596-018, Invitrogen™, USA) according to the manufacturer’s instructions. Reverse transcription was performed using the ABScript III RT Master Mix (RK20428, ABclonal, China). The relative RNA expression level was quantified via qPCR using Universal SYBR Green Fast qPCR Mix (RK21203, ABclonal, China) and gene-specific primers, with primer sequences provided in **Supplementary Table S1**. Protein expression level was analyzed by Western-blot (WB). Proteins from cell samples were separated using 10% SDS-polyacrylamide gel electrophoresis (SDS-PAGE) and transferred onto polyvinylidene fluoride (PVDF) membranes (IPVH00010, Sigma, USA). To prevent nonspecific binding, the membranes were blocked at room temperature with 5% nonfat milk for 2 hours. Subsequently, the membranes were incubated overnight at 4°C with primary antibodies. The following day, membranes were incubated at room temperature for 1 hour with horseradish peroxidase-conjugated goat anti-rabbit or anti-mouse IgG secondary antibodies (E030110-01, E030120-01, EarthOx, USA). Protein signals were detected and recorded using chemiluminescence.

### 2.10 Immunohistochemical staining

The immunohistochemical (IHC) staining procedure for patient tissue sections was performed following a previously established protocol (39). The reagents used included Triton X-100, immunohistochemical blocking buffer (P0102, Beyotime, China), antifade mounting medium (Solarbio, China), and anti-P2RY6 antibody (107142-T44, SinoBiological, China). Only pancreatic tissue samples with well-preserved morphology were selected for subsequent analysis. Staining intensity was evaluated using a 4-point scale: 0 (no staining), 1 (weak staining), 2 (moderate staining), and 3 (strong staining). The percentage of positively stained tumor cells was scored as follows: 1 (<10%), 2 (10–35%), 3 (35–70%), and 4 (>70%). The immunoreactive score (IRS) was calculated by multiplying the staining intensity score by the positive tumor cell proportion score. IRS scores ranging from 0 to 6 were classified as low expression, while scores of 6 or higher were categorized as high expression.

### 2.11 Lentiviral production and transduction

To establish stably transfected cell lines with P2RY6 overexpression, lentiviral vectors containing the P2RY6 sequence (pLV3-CMV-3×FLAG-P2RY6 (human)-Puro) or P2RY6-shRNA sequence (pLKO.1-U6-shRNA-Puro; shRNA sequence listed in **Supplementary Table S1**) were each co-transfected with packaging plasmids (psPAX2 and pMD2.G) into 293T cells. Following transfection, the viral supernatants were collected, concentrated, and subsequently used to infect Capan-1 and MIA-PaCa-2. To select for stably transfected cells, the infected cells were cultured in medium containing puromycin for one week, resulting in the generation of cell lines with stable overexpression or knockdown of P2RY6.

### 2.12 Cellular functional assays/

MIA-PaCa-2 and Capan-1 were seeded at a density of 1,500 cells per well in 96-well plates. Cell proliferation was assessed at 0, 24, 48, 72, and 96 hours by adding 10 μL of Cell Counting Kit-8 (CCK8, C0038, Beyotime, China) solution to each well, followed by a 2-hour incubation. Absorbance was measured at 450 nm. For the colony formation assay, cells were seeded at a density of 800 cells per well in 6-well plates and cultured for 10–14 days. Colonies were fixed with 4% paraformaldehyde and stained with 0.1% crystal violet for 10 minutes, and colony areas were quantified. To evaluate DNA replication activity, MIA-PaCa-2 and Capan-1 were incubated with 20 μM EdU (CX003, Cellorlab, China) solution for 2 hours. Cells were then fixed with 4% paraformaldehyde for 20 minutes at room temperature, and the proportion of EdU-positive cells was calculated. For drug sensitivity testing, cells were seeded at a density of 2,500 cells per well in 96-well plates and treated with varying concentrations of MRS-2578, with 0.1% DMSO serving as the control group. Cell viability was subsequently evaluated. In the migration assay, cells were serum-starved for 24 hours, and a linear wound was created using a 20 μL pipette tip. Wound closure was imaged at 0, 24, and 48 hours, and the change in wound area was quantified. For the Transwell invasion assay, 5 × 10^4^ MIA-PaCa-2 or 2.5 × 10^4^ Capan-1 cells were suspended in serum-free medium and seeded into the upper chambers of Transwell inserts coated with a 1:5 Matrigel (356234, Corning, China): PBS solution. After 36 hours, the invaded cells on the lower surface were fixed with 4% paraformaldehyde, stained with 0.1% crystal violet, and counted.

### 2.13 Cell cycle and apoptosis analysis

Cell cycle analysis was performed using a Cell Cycle and Apoptosis Analysis Kit (C1052, Beyotime, China) according to the manufacturer’s instructions. Red fluorescence signals were detected via flow cytometry with an excitation wavelength of 488 nm, and a minimum of 10,000 events was recorded for each sample. For apoptosis analysis, the Annexin V-FITC Apoptosis Detection Kit (C1062L, Beyotime, China) was employed. Flow cytometry was used to detect green fluorescence from Annexin V-FITC staining and red fluorescence from propidium iodide (PI) staining, with at least 10,000 events analyzed per sample. All data were processed and analyzed using FlowJo software.

### 2.14 Macrophage differentiation and tumor cell co-culture assay

THP-1 cells were seeded into the lower compartments of 6-well Transwell plates (Corning 3412) at a concentration of 1 × 10^5^ cells/mL and exposed to 80 ng/mL phorbol 12-myristate 13-acetate (PMA) for 12 hours to promote adherence and differentiation into M0 macrophages. After incubation, non-adherent cells and residual PMA were removed by PBS rinsing. Tumor cells, pretreated according to experimental requirements, were then added to the upper compartments at a 5:1 tumor-to-macrophage ratio and co-cultured for 24 hours. Following the co-culture period, macrophages were collected for total RNA extraction and analyzed by RT-qPCR to determine their polarization status. M1-like pro-inflammatory phenotype was defined by increased mRNA levels of CD80, CD86, and iNOS, while M2-like anti-inflammatory phenotype was indicated by higher expression of CD163, CD206, and Arg-1.

### 2.15 Subcutaneous tumor xenograft model in nude mice

For the xenograft experiments, 4-week-old male BALB/c athymic nude mice were housed in laminar flow cabinets under specific pathogen-free (SPF) conditions, with unrestricted access to food and water. To establish a PDAC xenograft model, 1×10^6^ MIA-PaCa2 or Capan-1 cells suspended in 100 µL PBS were subcutaneously injected into the right axilla of nude mice. Beginning on day 7 post-inoculation, mice were randomly assigned to receive intraperitoneal injections of either physiological saline or MRS-2578 every three days, with six mice per group. MRS-2578 was administered at a dose of 2 mg/kg, which has been reported as safe in previous studies (40). Tumor length and width (in millimeters) were measured using calipers on the day of drug administration and every three days thereafter. Tumor volume was calculated using the formula: (length × width²)/2. After the completion of intraperitoneal injections, all mice were euthanized, and subcutaneous tumors were excised and weighed. Tumor volumes and weights were presented as mean ± standard deviation. The number of animals per group was determined based on prior literature and preliminary studies to ensure sufficient statistical power for detecting significant differences in tumor growth. Treatment of mice and sample collection were blinded. All *in vivo* experiments were conducted in strict accordance with the Guide for the Care and Use of Laboratory Animals (National Institutes of Health, USA) and were approved by the Institutional Animal Care and Use Committee.

### 2.16 RNA sequencing

Total RNA was extracted from the samples using Trizol reagent, following the manufacturer’s protocol for isolation and purification. RNA quality control was performed using NanoDrop ND-1000 and Bioanalyzer 4200 to assess RNA concentration, purity, and integrity (concentration >20 ng/μL, RIN > 6.0, OD260/280 > 1.8). Poly(A) mRNA was purified using VAHTS mRNA Capture Beads 2.0, followed by fragmentation, cDNA synthesis, and library construction using the VAHTS Universal V8 RNA-seq Library Prep Kit. The target fragment size was selected to be 300 bp ± 50 bp. High-throughput paired-end sequencing (PE150) was performed on the Illumina Novaseq™ Xplus platform. The sequencing data were subjected to quality control using the fastp software (removing adapter sequences, low-quality reads, etc.) and then aligned to the human reference genome (Homo sapiens, GRCh38) using HISAT2. Transcript assembly and FPKM quantification were conducted using StringTie, and differential expression analysis was performed using the R “edgeR” package with the thresholds |log2FC| > 1 and p < 0.05.

### 2.17 Statistical analysis

Statistical analyses were performed using R software (v4.3.1) and GraphPad Prism software (v9). The p-value of less than 0.05 was considered statistically significant for all analyses.

## 3 Results

### 3.1 Construction and evaluation of prognostic features based on efferocytosis-related differential genes in PDAC

**Figure 1:**
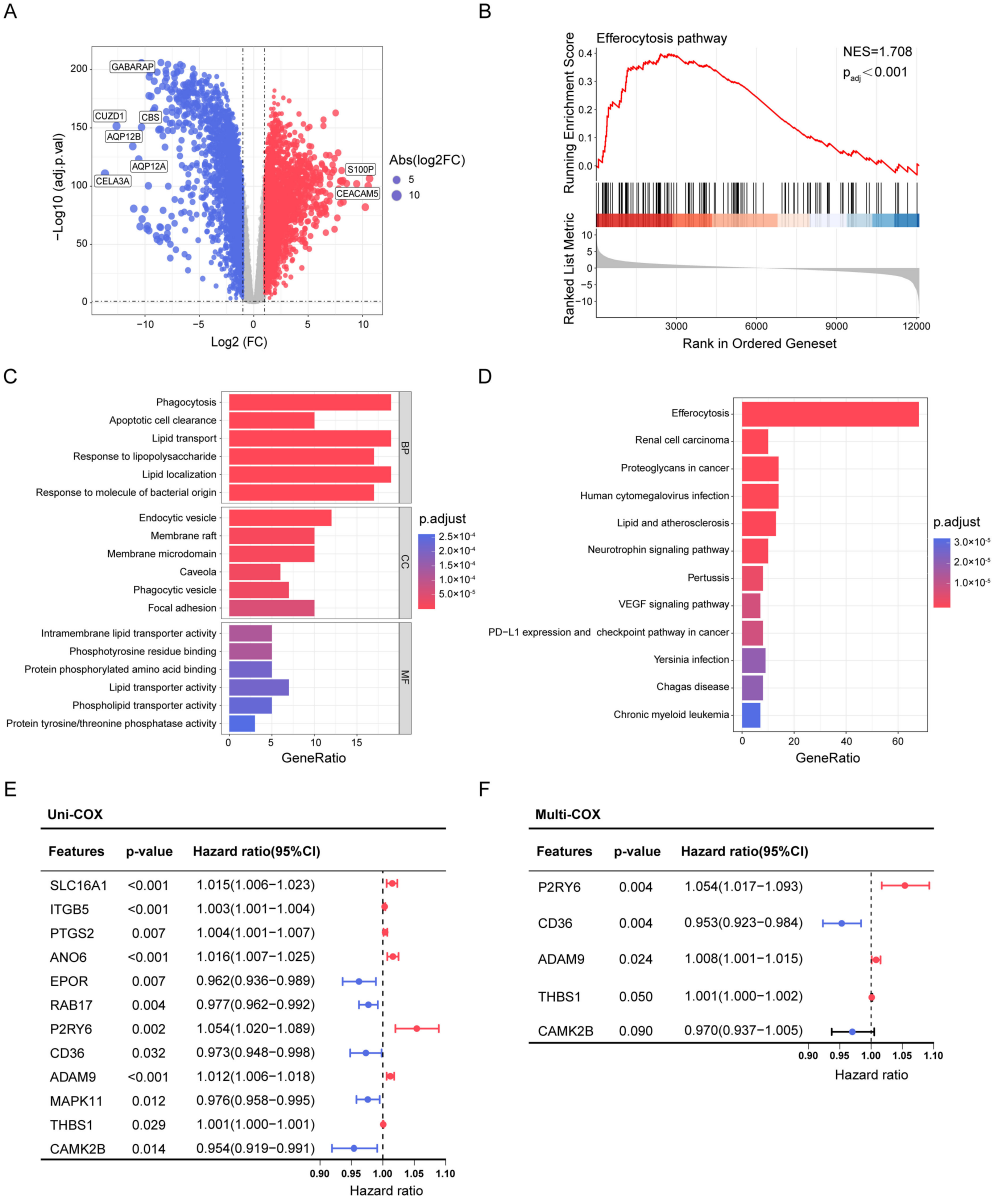
Screening, Functional Enrichment, and Prognostic Analysis of DE-ERGs in PDAC. **(A)** The volcano plot based on adjusted p-value and FC shows the DEGs (n = 5,871) between tumor and normal tissue. Red dots represent significantly upregulated genes, while blue dots indicate significantly downregulated genes (|log2(FC)| > 1 and adjusted p < 0.05). **(B)** GSEA plot showing significant activation of the KEGG efferocytosis pathway in PDAC (NES = 1.708, p < 0.001). The red curve indicates the running enrichment score, and black bars represent the positions of efferocytosis-related genes in the ranked gene list. **(C, D)** GO functional enrichment **(C)** and KEGG pathway analysis **(D)** reveal the biological functions and pathways enriched by DE-ERGs. **(E, F)** The forest plot demonstrates the results of univariate **(E)** and multivariate **(F)** Cox regression analysis based on DE-ERGs.


To explore the potential critical role of efferocytosis in PDAC, we analyzed transcriptomic data from the TCGA and GTEx databases and identified 5,871 differentially expressed genes (DEGs) between tumor and normal pancreatic tissues (**Figure 1A**). Gene set enrichment analysis (GSEA) revealed significant enrichment of efferocytosis pathways in PDAC tissue (**Figure 1B**). Subsequently, by intersecting identified DEGs with the efferocytosis gene set, we obtained 68 DE-ERGs (**Supplementary Figure S1A, B**). Functional enrichment analysis indicated that these DE-ERGs were significantly enriched in phagocytosis, lipid transport, and tumor-associated immunosuppression pathways, highlighting their potential roles in PDAC metabolic reprogramming and immune regulation (**Figures 1C, D**). To identify core genes closely associated with prognosis in PDAC patients, we performed univariate Cox regression and LASSO regression analyses on the DE-ERGs (**Supplementary Figures S1C, D**, **Supplementary Table S2**), ultimately identifying 12 genes significantly correlated with OS (**Figure 1E**). Further multivariate Cox regression analysis refined three key prognostic genes: *ADAM9*, *P2RY6*, and *CD36* (p < 0.05) (**Figure 1F**)—which were used to construct a prognostic scoring model, EFFscore, with the following formula:

EFFscore=1.2247*exp[(0.0121)*ADAM9+(0.0621)*P2RY6+(−0.0363)*CD36]

**Figure 2:**
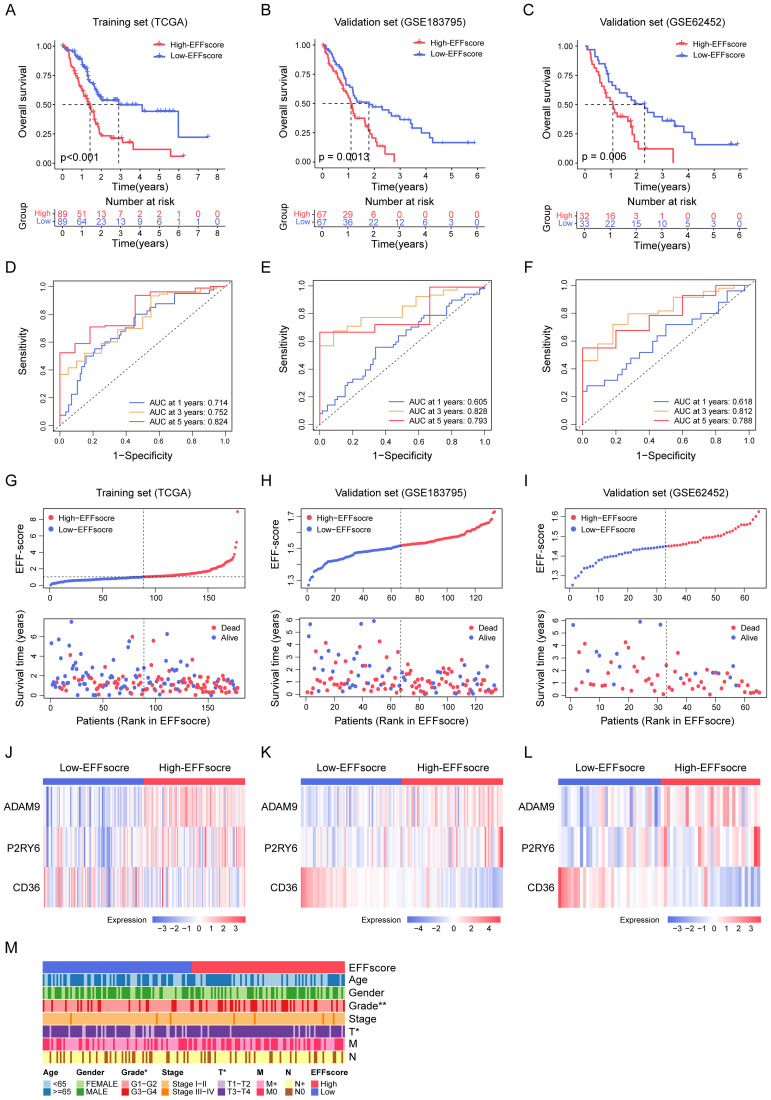
Stability and Predictive Performance of the EFFscore Model. **(A-C)** K-M survival curves show the survival of patients with high and low EFFscore in the TCGA-PAAD training set **(A)** and the GSE183795 **(B)** and GSE62452 **(C)** validation cohorts. **(D-F)** ROC curve analysis assesses the predictive performance of the EFFscore model for OS in the TCGA-PAAD training set **(D)** and the GSE183795 **(E)** and GSE62452 **(F)** validation cohorts. **(G-I)** Scatterplots display the patients’ survival status across increasing EFFscores in the TCGA-PAAD training set **(G)** and the GSE183795 **(H)** and GSE62452 **(I)** validation cohorts. **(J-L)** Heatmaps show the relative RNA expression levels of ADAM9, P2RY6, and CD36 across individual PDAC patient samples from the TCGA-PAAD training set **(J)** and the GSE183795 **(K)** and GSE62452 **(L)** validation cohorts. **(M)** Distribution of clinical and pathological characteristics between high and low EFFscore groups in the TCGA cohort. Each column represents a patient, annotated by age, gender, grade, stage, T, N, and M status. Statistical analyses were derived from Chi-square test and Fisher’s exact test, *p < 0.05, **p < 0.01.

To further evaluate the prognostic value of EFFscore, we calculated the EFFscore for PDAC patients in the training cohort (TCGA-PAAD; n = 178) and two validation cohorts (GSE183795; n = 134 and GSE62452; n = 65). Patients were stratified into High- and Low-EFFscore groups based on the median score. K-M analysis revealed that Low-EFFscore patients had significantly better prognosis in both training cohort (p < 0.001; **Figure 2A**) and validation cohorts (p = 0.001 and p = 0.006; **Figures 2B, C**). Time-dependent ROC analysis further confirmed the predictive power of EFFscore for 1-, 3-, and 5-year OS (**Figures 2D–F**). Additionally, EFFscore was found to be closely associated with survival time, survival status, and the expression patterns of the core genes *ADAM9*, *P2RY6* and *CD36* (**Figures 2G–L**). Clinicopathological analysis indicated that significant associations between EFFscore and tumor grade (p = 0.005) and T stage (p = 0.010) (**Figure 2M**, **Table 1**). Furthermore, cox regression identified age (p = 0.004) and EFFscore (p < 0.001) as independent prognostic factors (**Supplementary Figures S2A, B**). Based on these variables, we constructed a nomogram to predict OS, which displayed favorable calibration and discrimination (**Supplementary Figures S2C–G**). Together, these results establish EFFscore as a independently prognostic biomarker in PDAC, supporting its utility for individualized treatment planning.

**Table 1:** The correlation between EFF-score and clinicopathological characteristics of 176 TCGA patients.

Characteristics	Low-EFFscore (EFF-score<1.03)	High-EFFscore (EFF-score≥1.03)	P value
Age(n,%)			0.1300
<60	35(39.77%)	45(51.14%)	–
≥60	53(60.23%)	43(48.86%)	–
Gender			0.5446
Male	50(56.82%)	46(52.27%)	–
Female	38(43.18%)	42(47.73%)	–
Grade(n,%)			0.0047^**^
G_I-II_	71(80.68%)	54(61.36%)	–
G_III-IV_	17(19.32%)	34(38.64%)	–
TNM stage(n,%)			0.6995
I-II	85(96.59%)	84(95.45%)	
III-IV	3(3.41%)	4(4.54%)	
T stage(n,%)			0.0101^*^
T_1-2_	22(25.00%)	9(10.23%)	
T_3-4_	66(75.00%)	79(89.77%)	
M stage(n,%)			0.0693
M_0_	34(38.64%)	46(52.27%)	
M_+_	54(61.36%)	42(47.73%)	
N stage(n,%)			0.3099
N_0_	27(30.68%)	21(23.86%)	
N_+_	61(69.32%)	67(76.14%)	

(Chi-square test and Fisher’s exact test; *p < 0.05, **p < 0.01).

### 3.2 The association between EFFscore and genome variation, immune infiltration, and drug sensitivity in PDAC patients

Considering that types of genome variation and mutation burden significantly influence patient prognosis, we analyzed somatic mutations in PDAC patients stratified by EFFscore. Data derived from TCGA revealed that missense mutations and single nucleotide polymorphisms (SNPs) predominate in both High- and Low-EFFscore groups (**Supplementary Figures S3A, B**). However, High-EFFscore patients exhibit significantly higher mutation frequencies of *KRAS* (77% *vs*. 43%), *TP53* (77% *vs*. 38%), *CDKN2A* (25% *vs*. 10%), and *SMAD4* (24% *vs*. 19%) compared to their Low-EFFscore counterparts (**Supplementary Figures S3C, D**). Consistently, TMB was significantly elevated in High-EFFscore patients (p < 0.05; **Supplementary Figure S3E**), correlating with worse prognosis in these individuals (**Supplementary Figure S3F**). In addition, CNV analysis indicates that *P2RY6* predominantly displays gain-of-function mutations in High-EFFscore patients, whereas loss-of-function mutations dominate in Low-EFFscore patients (**Supplementary Figure S3G, H**), suggesting a distinctive role for P2RY6 in PDAC progression.

**Figure 3:**
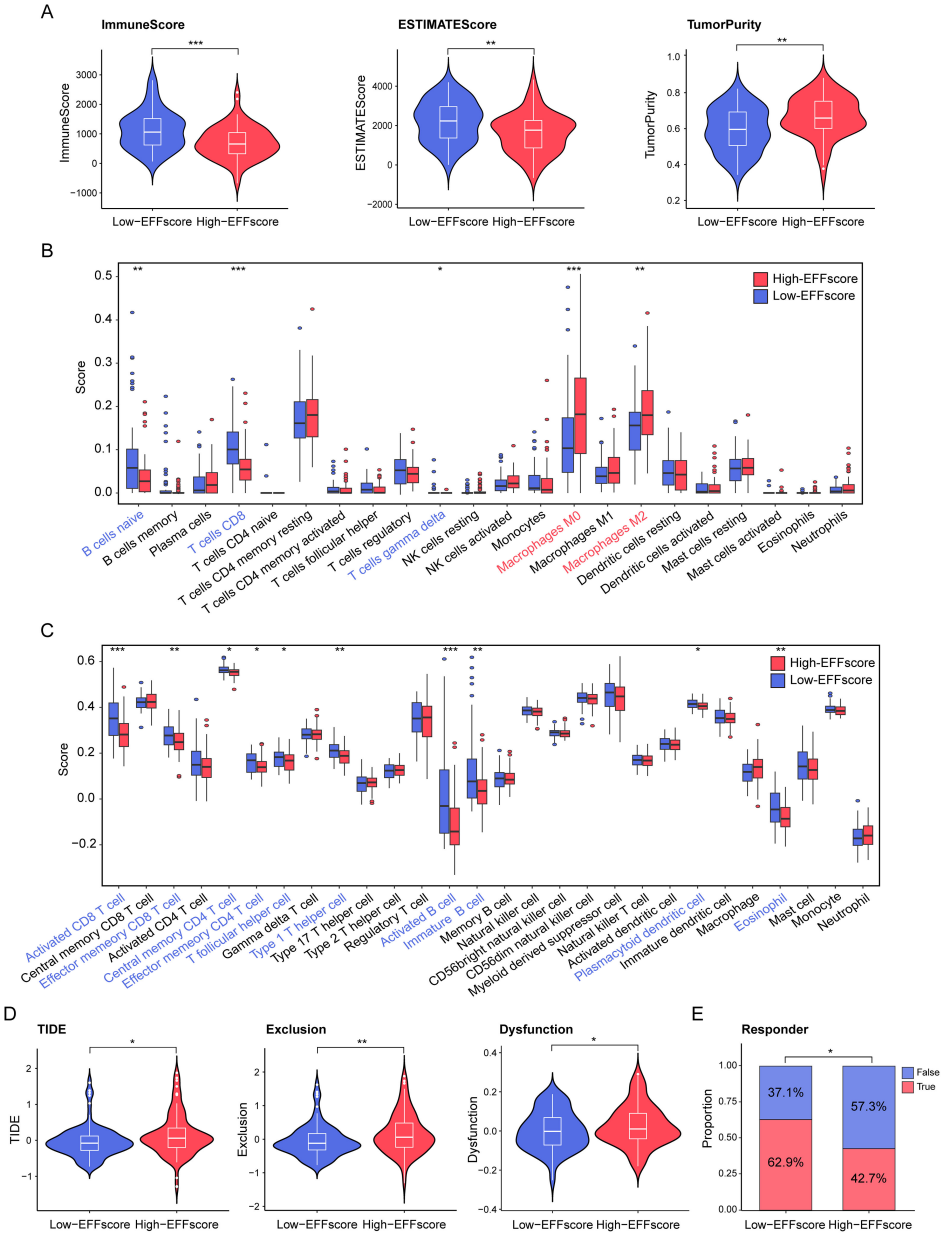
The difference in immune infiltration and immunotherapy response between High-and Low-EFFscore patients. **(A)** Comparison of the immune score, ESTIMATE score, and tumor purity between patients with high and low EFFscore. **(B, C)** Immune infiltration patterns between High- and Low-EFFscore patients were assessed by the ssGSEA **(B)** and the CIBERSORT algorithm **(C)**. **(D, E)** TIDE analysis assessing predicted immunotherapy response, including TIDE score, exclusion score, dysfunction score **(D)** and immunotherapy response **(E)**, with red indicating responders and blue indicating non-responders. Statistical analyses include Mann-Whitney U test **(A-D)** and Chi-square test **(E)**, *p < 0.05, **p < 0.01, ***p < 0.001.

Given the critical role of efferocytosis in promoting immunosuppression, we utilized three algorithms—ESTIMATE, CIBERSORT, and ssGSEA to assess the differences of TME composition in PDAC patients. The results demonstrated that Low-EFFscore patients exhibited lower tumor purity but higher immune and ESTIMATE scores, indicating a potentially more immunoactive microenvironment (**Figure 3A**). Specifically, these patients also exhibited higher abundances of immunoregulatory cells (e.g., plasmacytoid dendritic cell and follicular helper T cell), immune effector cells (e.g., Th1 cells, activated CD8^+^ T cell, and activated B cell), and immune memory cells (e.g., effector memory CD4^+^ and CD8^+^ T cell). In contrast, High-EFFscore patients exhibited greater macrophage infiltration—especially M2 phenotypes—consistent with efferocytosis-driven immunosuppressive (**Figures 3B, C**). Furthermore, TIDE algorithm revealed that High-EFFscore patients had significantly higher TIDE, immune exclusion and dysfunction scores, along with a reduced predicted response to immunotherapy (42.7% *vs*. 62.9%; **Figures 3D, E**). Collectively, these results underscore the immunosuppressive landscape of High-EFFscore patients and their limited sensitivity to current immunotherapy strategies.

Genome variation is known to tumor drug resistance through diverse mechanisms (41). To elucidate the association between EFFscore and drug sensitivity, we evaluated the response of PDAC patients to both conventional chemotherapies and targeted therapies. Based on the 2023 European Society for Medical Oncology (ESMO) guidelines (42), we selected chemotherapeutics (Gemcitabine, Oxaliplatin, Fluorouracil, Irinotecan, and Carboplatin), NOTCH inhibitor (MK-0752), TGF-β inhibitor (LY2157299), JAK inhibitor (Ruxolitinib), and tyrosine kinase inhibitors (Sorafenib, Sunitinib, Canertinib, and Masitinib) for drug sensitivity analysis. The results indicate that High-EFFscore patients, characterized by elevated genome variation level, exhibit increased resistance to conventional chemotherapeutics and tyrosine kinase inhibitors but enhanced sensitivity to MK-0752, LY2157299, and Ruxolitinib (**Supplementary Figures S4A–L**). These findings suggest that High-EFFscore patients may exhibit activation of the NOTCH, TGF-β, and JAK-STAT pathways.

### 3.3 The EFFscore is closely associated with the tumor evolution, and malignant biological characteristics of PDAC

**Figure 4:**
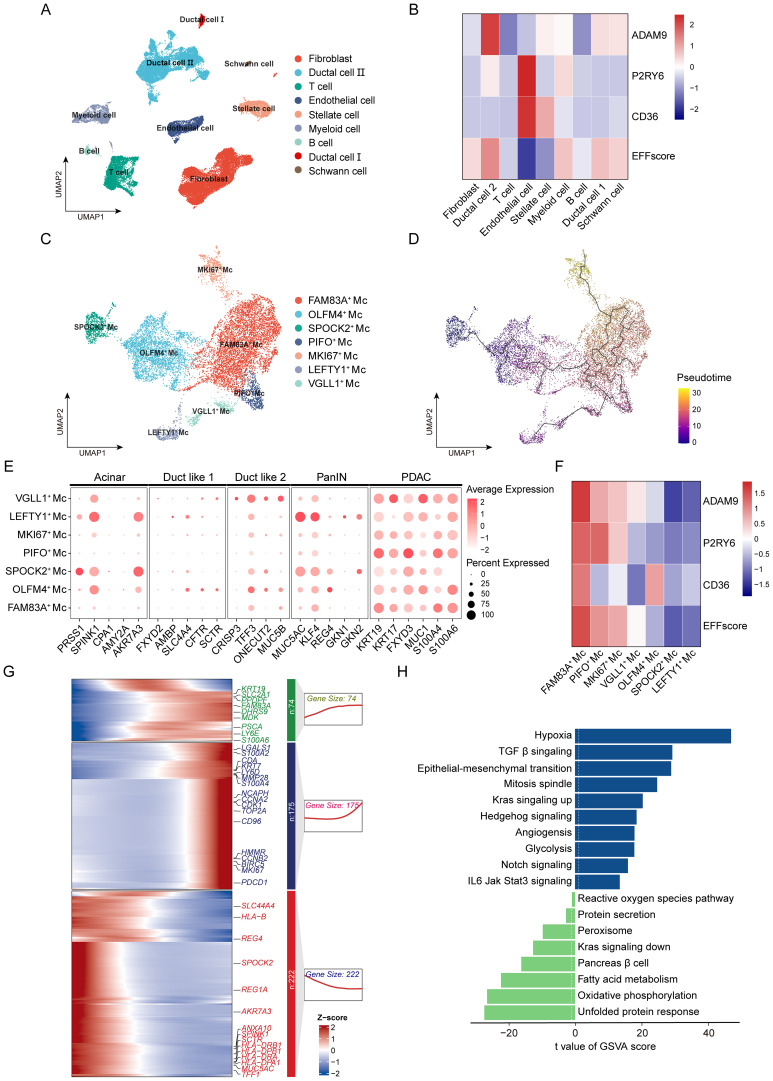
The distribution of EFFscore in cell clusters and its potential correspondence to tumor evolution stage. **(A)** The UMAP plot depicts the clustering distribution of major cell clusters within the dataset. **(B)** Heatmap plot shows the expression and distribution of *ADAM9*, *P2RY6*, *CD36* and EFFscore across different cell types. **(C)** The UMAP plot depicts the clustering of PDAC cells into distinct subclusters. **(D)** Pseudotime trajectory analysis reveals the dynamic evolution of PDAC cells, with cells colored according to pseudotime. **(E)** Bubble plot displays the expression of signature genes at different evolutionary stages of PDAC progression. Bubble size represents the proportion of cells expressing marker genes, while color indicates the average expression of the genes within the cells. **(F)** Heatmap plot shows the expression and distribution of *ADAM9*, *P2RY6*, *CD36*, and EFFscore across the PDAC cell subclusters. **(G)** Pseudotime heatmap plot shows dynamic changes in gene expression along the evolutionary trajectory, with the x-axis representing pseudotime progression. **(H)** GSVA analysis highlighting pathway enrichment differences between High- and Low-EFFscore subgroup, elucidates their functional characteristics.

To further elucidate the distribution of EFFscore within the TME, we analyzed scRNA-seq dataset from GSE194247 and GSE235449, identifying nine major cell clusters (**Figure 4A**, **Supplementary Figures S5A, B**). Notably, two distinct ductal cells subtypes were identified. Type II ductal cells exhibited high expression of PDAC-associated malignant markers (e.g., *MUC1*, *FXYD3*, and *KRT19*), whereas Type I ductal cells expressed markers characteristic of normal pancreatic ducts (e.g., *AMBP*, *SLC4A4*, and *FXYD2*) (**Supplementary Figure S5B**). Furthermore, CNV analysis revealed significantly elevated CNV levels in Type II ductal cells (**Supplementary Figure S5D**), supporting their identity as malignant PDAC cells. Among all clusters, Type II ductal cells exhibited the highest EFFscore, markedly higher than Type I ductal cells (**Figure 4B**). Consistent results were obtained from another single-cell dataset (GSA: CRA001160) (**Supplementary Figures S6A–D**).

Given the marked tumor heterogeneity within PDAC, we further subdivided Type II ductal cells into seven distinct cell subclusters and characterized their gene expression profiles (**Figure 4C**, **Supplementary Figure S5C**). Referring to the hallmark genes delineated by Daniel et al. for PDAC progression (**Figure 4E**) (43), we hypothesized that these subclusters correspond to distinct evolutionary stages of PDAC. For instance, *SPOCK2*^+^ Mc and *LEFTY1*^+^ Mc subclusters exhibited high expression of PanIN-associated markers (*MUC5AC*, *KLF4*), whereas *VGLL1*^+^ Mc subclusters expressed Duct-like 2 markers (*CRISP3*, *MUC5B*, and *ONECUT2*). In contrast, subclusters such as *FAM83A*^+^ Mc, *PIFO*^+^ Mc, and *MKI67*^+^ Mc were enriched for advanced PDAC marker (*KRT19*, *FXYD3*, and *S100A6*) [36]. Pseudotime trajectory analysis further revealed a hierarchical differentiation pattern, in which early subgroups (*SPOCK2*^+^Mc/*LEFTY1*^+^Mc) progress through intermediate states (*OLFM4*^+^Mc/*VGLL1*^+^Mc) to late-stage subgroups (*PIFO*^+^Mc/*FAM83A*^+^Mc/*MKI67*^+^Mc) (**Figure 4D**). During this progression, dynamic transcriptional remodeling occurs: expression of MHC molecules (*HLA-B*, *HLA-DRA*, *HLA-DRB1*) and normal pancreatic genes declines, while cell proliferation (*MKI67*), anti-apoptotic (*BIRC5*), and drug resistance-related genes (*CDA*) progressively increase (**Figure 4G**). Notably, EFFscore also increased progressively along the pseudotime trajectory, consistent with the malignant evolution from early to late subclusters (**Figure 4F**).

To further elucidate the malignant characteristics associated with EFFscore, we defined *PIFO*^+^ Mc, *FAM83A*^+^ Mc, and *MKI67*^+^ Mc as the High-EFFscore subgroup, while the remaining subclusters comprising the Low-EFFscore subgroup. High-EFFscore subgroup exhibited significantly elevated CNV levels (**Supplementary Figures S5E, F**), indicating increased genomic instability. GSVA revealed enrichment of glycolysis and hypoxia signatures in the High-EFFscore subgroup (**Figure 4H**). In contrast, Low-EFFscore subgroup was enriched in oxidative phosphorylation and fatty acid metabolism pathways. Moreover, High-EFFscore subgroup exhibited elevated mitotic activity, angiogenic potential, and epithelial-mesenchymal transition (EMT), accompanied by activation of KRAS, TGF-β, NOTCH, Hedgehog, and JAK-STAT pathways. Conversely, the Low-EFFscore subgroup exhibited pronounced activation of unfolded protein response (UPR) and retained partial pancreatic secretory functions.

In summary, EFFscore is closely associated with the evolutionary state of tumor cells. Subgroup with high EFFscore exhibits malignant characteristics strongly correlated with lower tumor differentiation, supporting a potential link between EFFscore and tumor grade.

### 3.4 Extensive interactions between high-EFFscore subgroup and microenvironmental components promote tumor progression

**Figure 5:**
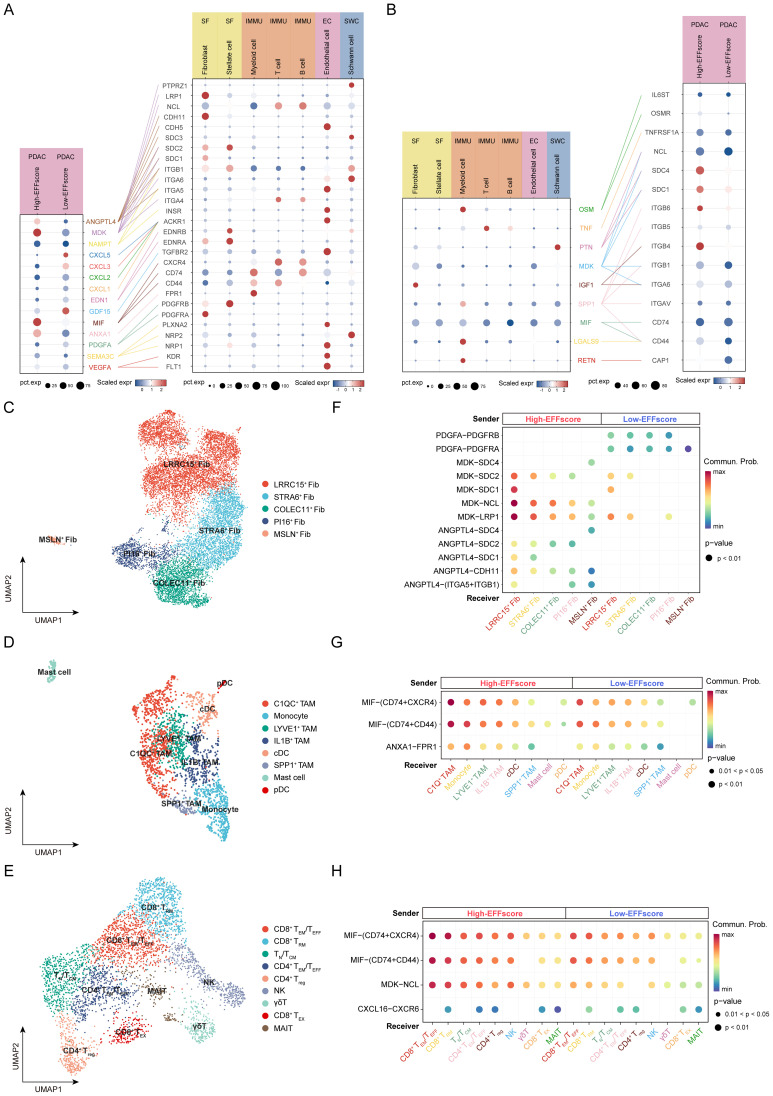
Ligand-Receptor Interaction Network Between Distinct EFFscore Subgroups and Microenvironmental Cell. **(A, B)** Dot plot shows potential ligand-receptor interactions between distinct EFFscore subgroups and microenvironmental cell, with PDAC cells acting as signal senders **(A)** and receivers **(B)**. Bubble size represents the proportion of cells expressing marker genes, while color indicates the average expression of the genes within the cells. **(C-E)** UMAP plot depicts the clustering distribution of fibroblast sub clusters **(C)**, myeloid cell subclusters **(D)**, and T cell subclusters **(E)**. **(F-H)** Ligand-receptor interactions between distinct EFFscore subgroups (donors) and fibroblasts **(F)**, myeloid cells **(G)**, and T cells **(H)** as receptors. Dot color represents interaction probability, and dot size indicates the statistical significance (p-value).

Tumor progression is driven not only by intrinsic genetic alterations but also by extrinsic regulation from the TME. We applied CellChat analysis to elucidate the differences in ligand-receptor interactions between EFFscore subgroups and TME components. Fibroblasts, myeloid cells, and T cells emerged as the primary interactors with PDAC cells, with the High-EFFscore subgroup exhibiting stronger interaction intensity (**Supplementary Figures S7A, B**). Notably, High-EFFscore subgroup exhibited enhanced signaling activity involving Midkine (MDK), Angiopoietin-like 4 (ANGPTL4), Macrophage migration inhibitory factor (MIF), and Annexin A1 (ANXA1), suggesting their potential roles in remodeling the TME (**Figure 5A**).

To further investigate the source of these signals, we performed dimensionality reduction and subclustered fibroblasts, myeloid cells, and T cells were based on their communication profiles (**Supplementary Figures S8A–C**, **Figures 5C-E**). Among fibroblast subsets, myofibroblasts (myCAFs), specifically *LRRC15*^+^ Fib, and *STRA6*^+^ Fib emerged as the primary interactors with PDAC cells. High-EFFscore subgroup likely interacts with fibroblasts through MDK–Syndecan (SDC)/Low-density lipoprotein receptor-related protein 1 (LRP1)/Nucleolin (NCL) and ANGPTL4–SDC/Cadherin 11 (CDH11)/Integrin alpha-5 (ITGA5) axis, whereas Platelet-derived growth factor A (PDGFA)-PDGFRA/PDGFRB interactions were more prominent in the Low-EFFscore subgroup (**Figure 5F**). Although the patterns of immune cell communication were broadly similar between the two subgroups, certain signaling pathways were differentially activated. High-EFFscore subgroup exhibited enhanced MIF-CD74 and MDK-NCL signaling activities, as well as enhanced interaction with myeloid cells mediated by ANXA1–formyl peptide receptor 1 (FPR1) (**Figure 5G**). Conversely, Low-EFFscore subgroup exhibited enriched CXCL16–CXCR6 signaling involving T cells (**Figure 5H**).

Subsequently, we reversed the direction of receptor-ligand interactions and assessed PDAC cells as signal recipients, identifying myeloid cells as the dominant signal senders (**Figure 5B**). Among them, monocytes and specific macrophage subsets preferentially interacted with High-EFFscore subgroup through Secreted phosphoprotein 1 (SPP1)–CD44/(ITGAV+ITGB), Resistin (RETN)–adenylate cyclase-associated protein 1 (CAP1) and Oncostatin M (OSM)–(OSMR+IL6ST) axes (**Supplementary Figure S7C**).

In summary, these findings delineate the complex signaling patterns between PDAC cells and microenvironmental cells. The characteristic interactions of the high EFFscore subgroup with TME components coupled with the activation of oncogenic pathways within tumor cells, may cooperatively drive PDAC progression.

### 3.5 Elevated expression of P2RY6 in PDAC correlates with poor prognosis

**Figure 6:**
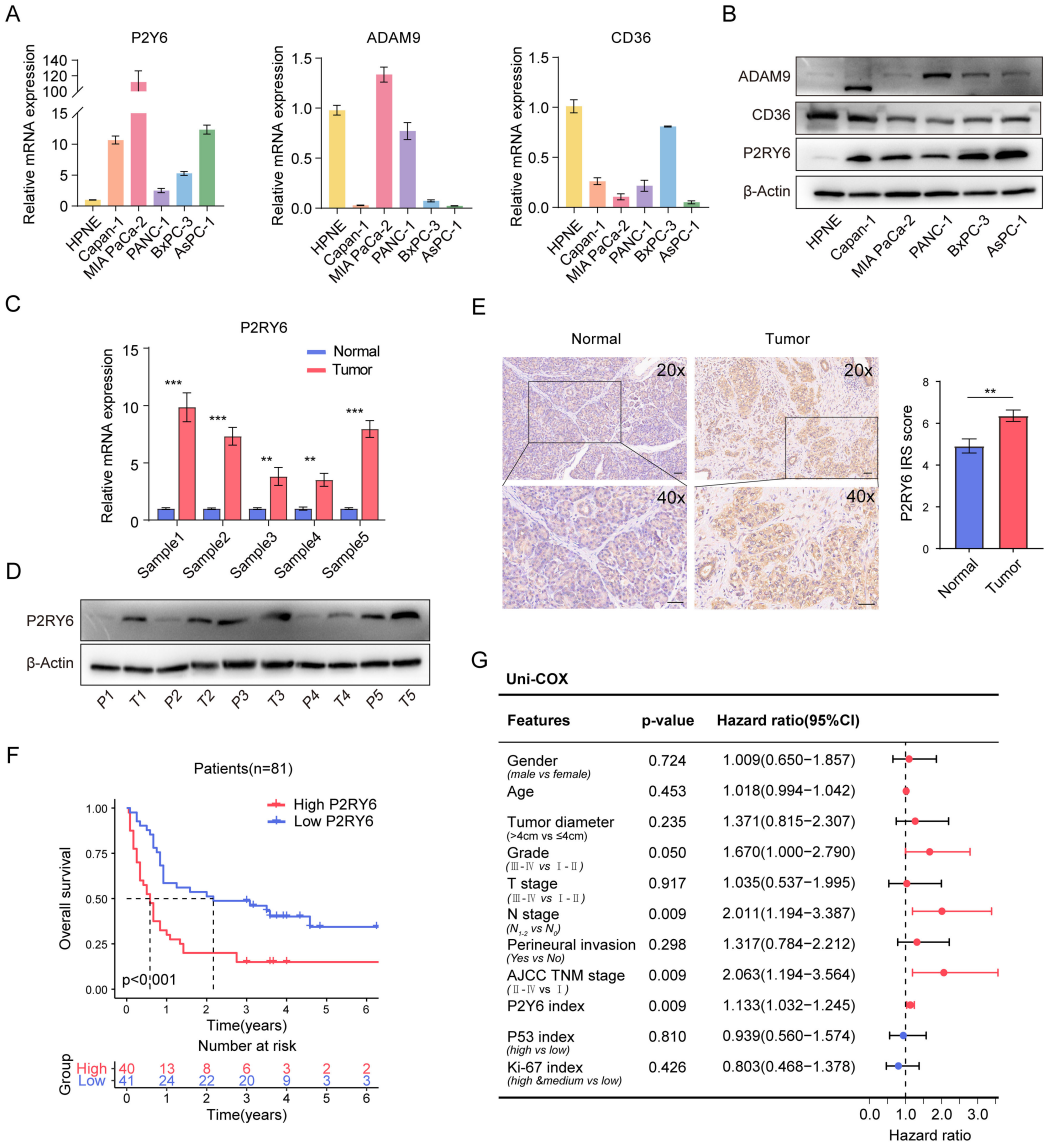
Upregulation of P2RY6 in PDAC and Its Role in Promoting PDAC Progression. **(A, B)** RT-qPCR **(A)** and WB analysis **(B)** demonstrates the expression of three genes in normal ductal cells and PDAC cell lines. **(C, D)** The expression of P2RY6 in five pairs of PDAC tissues and their corresponding adjacent normal tissues. **(E)** Representative IHC staining images of P2RY6 in primary PDAC and adjacent normal tissues, alongside quantified IRS scores comparing P2RY6 expression between tumor (N = 81) and normal tissues (N = 44), presented as mean ± SEM (scale bars: wound healing assay, 25μm). **(F)** K-M survival analysis shows the survival curves of 81 PDAC patients with high (IRS ≥ 6, N = 40) and low (IRS < 6, N = 41) P2RY6 expression. **(G)** Forest plot summarizes the results of univariate Cox regression analysis based on clinicopathological data from 81 PDAC patients, highlighting significant factors and their prognostic value. Statistical analyses include Unpaired t-test **(C)**, Chi-square test **(G)**, error bars represent standard deviation (SD) in **(A, C)** and standard error of the mean (SEM) in **(E)**, **p < 0.01, ***p < 0.001.

We first assessed the expression level of ADAM9, P2RY6, and CD36 in normal pancreatic ductal epithelial cells (HPNE) and various PDAC cell lines (Capan-1, MIA-PaCa2, PANC-1, BxPC-3, and AsPC-1). RT-qPCR and WB analyses revealed that P2RY6 expression was markedly upregulated in both PDAC cell lines and tumor tissues compared to normal controls (**Figures 6A–D**). Consistently, IHC staining of clinical specimens demonstrated significantly higher P2RY6 expression in tumor tissue than normal pancreatic tissue, which was strongly associated with reduced OS (**Figures 6E–G**). Moreover, P2RY6 expression positively correlated with tumor grade, showing higher P2RY6 expression in poorly differentiated tumors (**Table 2**). Together, these findings collectively indicate that aberrant P2RY6 upregulation is closely associated with PDAC progression and poor prognosis.

**Table 2:** The correlation between P2RY6 abundance and clinicopathological characteristics of 81 PDAC patients.

Characteristics	Low P2RY6 (IRS<6)	High P2RY6 (IRS≥6)	P value
Age(n,%)			0.725
<60	19(46.34%)	16(40.00%)	–
≥ 60	22(53.66%)	24(60.00%)	–
Gender			0.295
Male	22(53.66%)	27(67.50%)	–
Female	19(46.34%)	13(32.50%)	–
Tumor diameter(n,%)			0.919
≤ 4cm	23(56.10%)	21(55.25%)	–
>4cm	18(43.90%)	19(47.50%)	–
Grade(n,%)			0.011^*^
I-II	27(65.85%)	14(35.00%)	–
III-IV	14(34.15%)	26(65.00%)	–
AJCC TNM stage(n,%)			0.896
I	18(43.90%)	16(40.00%)	
II-IV	23(56.10%)	24(60.00%)	
T stage(n,%)			0.434
T_1-2_	31(75.61%)	34(85.00%)	
T_3-4_	10(25.39%)	6(15.00%)	
N stage(n,%)			0.584
N_0_	24(58.54%)	20(50.00%)	
N_1-2_	17(41.46%)	20(50.00%)	
Perineural invasion (n,%)			1.000
NO	25(61.76%)	25(62.50%)	
Yes	16(39.24%)	15 (37.50%)	
Ki67 index(n,%)			0.312
<10%	29(70.73%)	23(57.50%)	
≥10%	12(29.27%)	17(42.50%)	
P53 index(n,%)			0.747
<50%	24(58.54%)	21(52.50%)	
≥50%	17(41.46%)	19 (47.50%)	

AJCC stage American Joint Committee on Cancer stage (Chi-square test; *p < 0.05).

### 3.6 P2RY6 inhibition suppresses malignant biological behavior of PDAC

**Figure 7:**
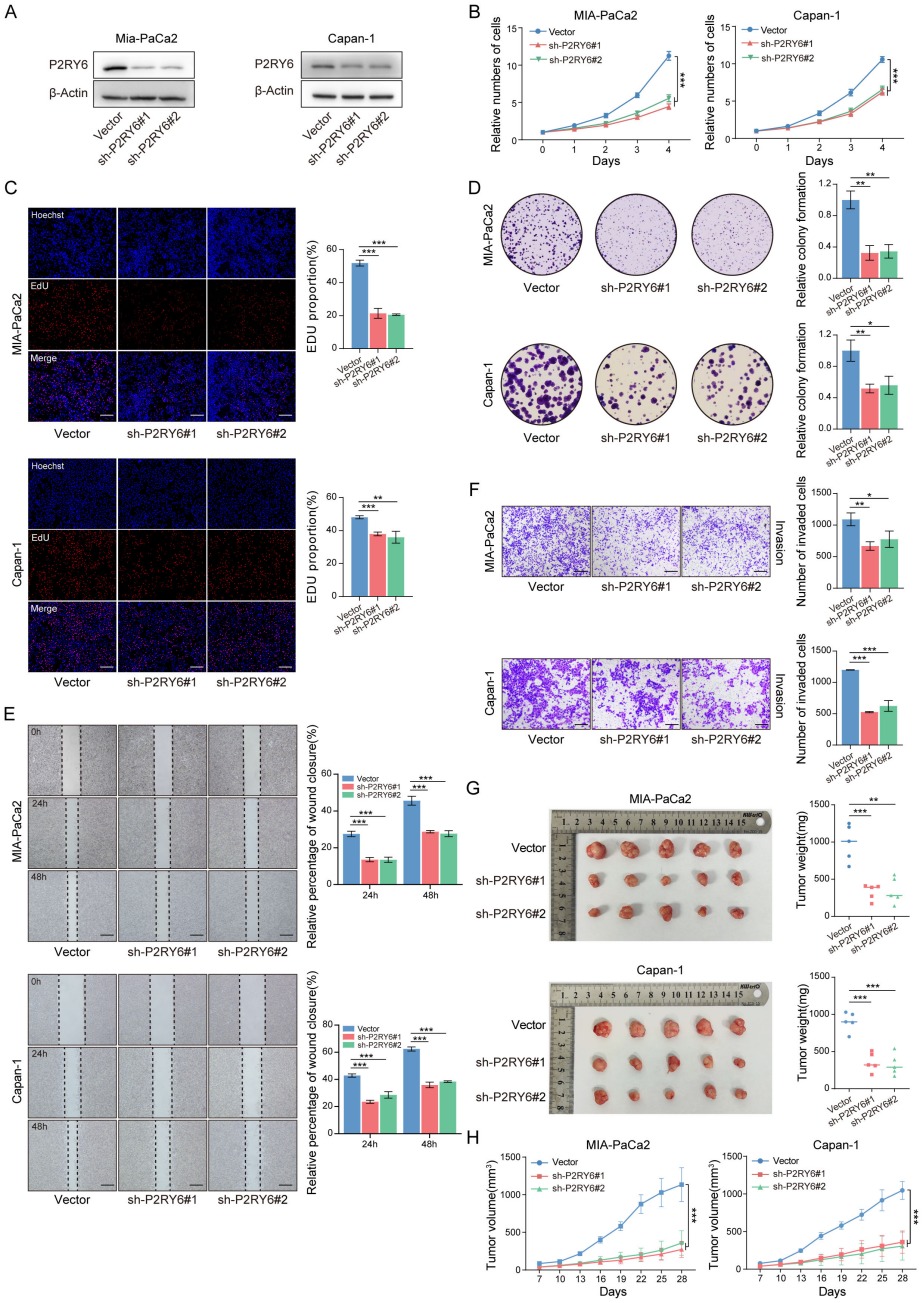
P2RY6 Knockdown Significantly Inhibits the Malignant Biological Behavior of PDAC. **(A)** P2RY6 protein level in MIA-PaCa2 and Capan-1 transfected with vector or sh-P2RY6#1/2. **(B-F)** Analysis of malignant biological behaviors in vector or sh-P2RY6#1/2-transfected MIA-PaCa2 and Capan-1, including proliferation curves **(B)**, representative images of EdU staining **(C)**, colony formation assays **(D)**, wound healing assays **(E)**, and Transwell invasion assays **(F)**. (Scale bars: EdU staining, 30 μm; wound healing, 12 μm; Transwell invasion, 30 μm) **(G, H)** Subcutaneous tumor xenograft model shows the tumor size **(G)** and tumor volume changes **(H)** of MIA-PaCa2 and Capan-1 transfected with vector or sh-P2RY6#1/2. Statistical analyses were derived from Unpaired t-test, error bars represent SD based on three independent experiments (n = 3), *p < 0.05, **p < 0.01, ***p < 0.001.

To investigate the biological functions of P2RY6 in PDAC, we established stable P2RY6 knockdown in Capan-1 and MIA-PaCa2 cell lines (**Figure 7A**). CCK-8 and colony formation assays demonstrated that P2RY6 knockdown significantly inhibited the proliferative capacity of Capan-1 and MIA-PaCa2 (**Figures 7B, D**). Furthermore, EdU assays revealed marked impairment of DNA replication in P2RY6- knockdown cells (**Figure 7C**). Wound healing and Transwell invasion assays demonstrated that P2RY6 knockdown significantly suppressed the migratory and invasive abilities of these cells (**Figures 7E, F**). Consistently, tumor growth was significantly inhibited in tumor xenograft model with P2RY6 knockdown (**Figures 7G, H**). These findings highlight that P2RY6 is essential for maintaining the malignant biological behaviors of PDAC cells, serving as a promising therapeutic target.

**Figure 8:**
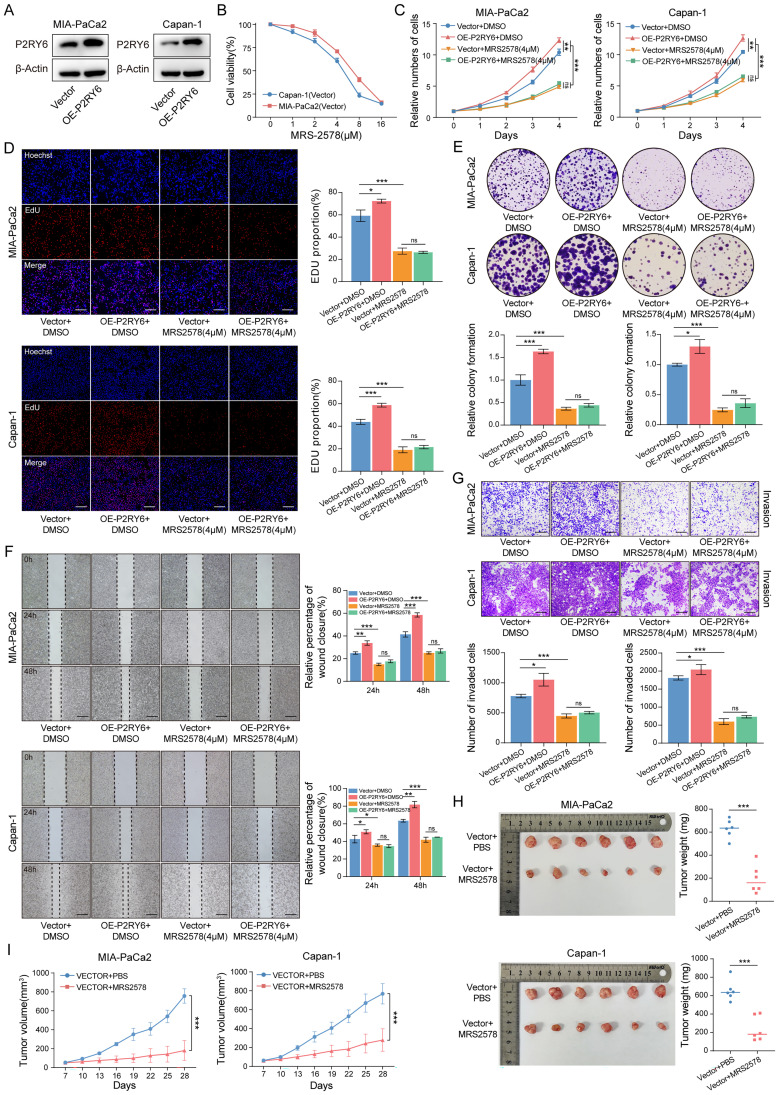
The P2RY6 inhibitor MRS-2578 can reverse the pro-cancer effects mediated by the overexpression of P2RY6. **(A)** P2RY6 protein level in MIA-PaCa2 and Capan-1 transfected with vector or P2RY6 overexpression (P2RY6-OE). **(B)** Sensitivity analysis of MIA-PaCa2 and Capan-1 to MRS-2578. **(C-E)** Analysis of malignant biological behaviors in vector or sh-P2RY6#1/2-transfected MIA-PaCa2 and Capan-1 treated with MRS-2578 or DMSO, including proliferation curves **(C)**, representative images of EdU staining **(D)**, colony formation assays **(E)**, wound healing assays **(F)**, and Transwell invasion assays **(G)**. (Scale bars: EdU staining, 30 μm; wound healing, 75 μm; Transwell invasion, 30 μm.). **(H, I)** Subcutaneous tumor xenograft model shows the tumor size **(H)** and tumor volume changes **(I)** of MIA-PaCa2 and Capan-1 treated with MRS-2578 or PBS. Statistical analyses were derived from Unpaired t-test, error bars represent SD based on three independent experiments (n = 3), ^ns^ p >0.05, *p < 0.05, **p < 0.01, ***p < 0.001.

To further validate whether P2RY6 directly promotes the malignant phenotype of PDAC cells, we generated P2RY6-overexpressing Capan-1 and MIA-PaCa2 (**Figure 8A**) and employed MRS-2578, a specific antagonist of the P2RY6 receptor, for targeted inhibition. Drug sensitivity analysis revealed that MRS-2578 exhibited significant inhibitory effects on PDAC cells at a concentration of 4 μM (**Figure 8B**). CCK-8, colony formation, and EdU assays confirmed that P2RY6 overexpression significantly promoted PDAC cell proliferation, whereas MRS-2578 treatment effectively abrogated this effect (**Figures 8C–E**). Similarly, wound healing and Transwell invasion assays demonstrated that P2RY6 overexpression promoted PDAC cell migration and invasion, both of which were substantially inhibited by MRS-2578 (**Figures 8F, G**). Consistently, treatment with MRS-2578 significantly reduced tumor burden in xenograft models (**Figures 8H, I**).

Considering the potential involvement of P2RY6 in immune evasion, we first stratified PDAC patients from TCGA database based on the median P2RY6 expression level and conducted TIDE algorithm analysis. Patients with high P2RY6 expression exhibited significantly elevated TIDE, dysfunction, and exclusion scores, along with a reduced predicted respond to immunotherapy (40.4% *vs*. 65.2%; **Supplementary Figures S9A, B**). Furthermore, macrophage co-culture assays revealed that the P2RY6 knockdown group exhibited a shift in macrophage polarization, with a decreased proportion of M2 macrophages and an increased proportion of M1 macrophages (**Supplementary Figure S9C**). These findings demonstrate that P2RY6 contributes to promoting an immunosuppressive microenvironment and reinforce its potential as a therapeutic target in PDAC.

### 3.7 P2RY6 knockdown exerts tumor-suppressive effects via ERS pathway and the MHC antigen presentation process

**Figure 9:**
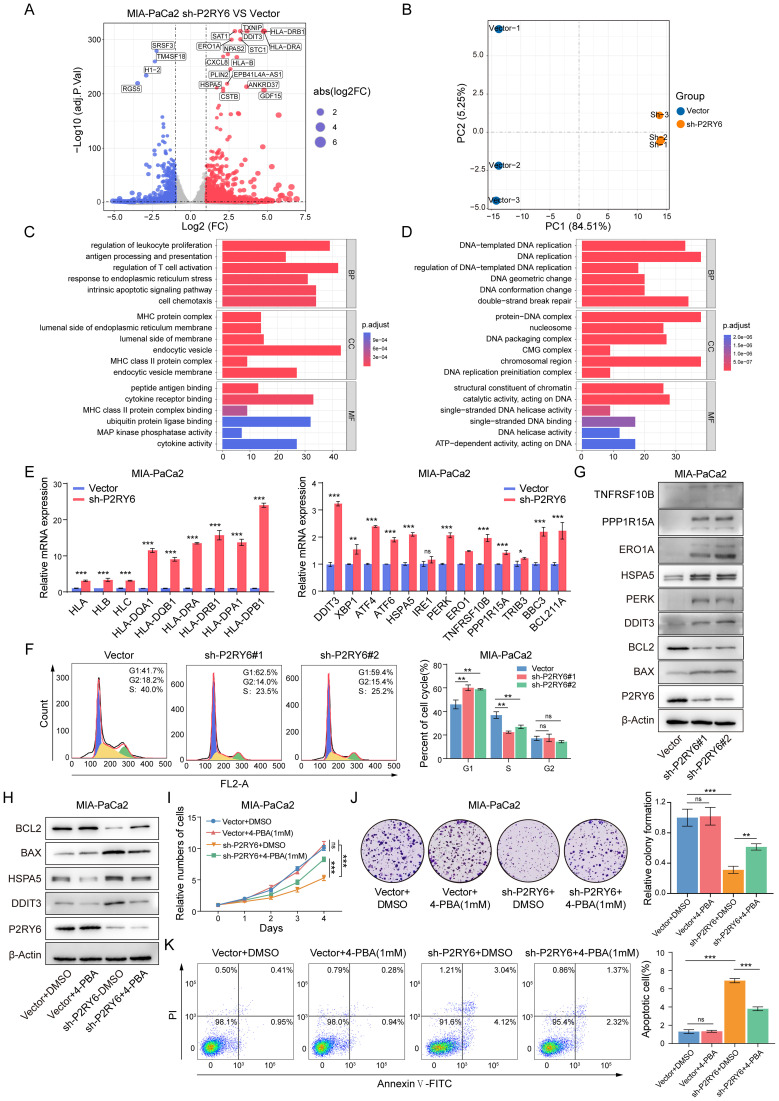
P2RY6 Knockdown Exerts Tumor-Suppressive Effects via ERS and MHC Antigen Presentation Process. **(A)** Volcano plot shows transcriptomic alterations between vector or sh-P2RY6#1-transfected MIA-PaCa2. Red dots represent significantly upregulated genes, and blue dots represent significantly downregulated genes (|log_2_(FC)| > 1 and adjusted p < 0.05). **(B)** PCA demonstrates clustering distributions of samples between vector and sh-P2RY6#1-transfected MIA-PaCa2. **(C-D)** GO enrichment analysis shows functional enrichment of upregulated **(C)** and downregulated **(D)** genes. **(E)** RT-qPCR validation of RNA expression for genes associated with MHC antigen presentation and ERS in MIA-PaCa2. **(F)** Flow cytometric analysis of cell cycle distribution in vector or sh-P2RY6#1/2-transfected MIA-PaCa2. **(G)** The expression of ERS-related proteins in vector or sh-P2RY6-transfected MIA-PaCa2. **(H)** ERS and apoptosis-related protein in vector or sh-P2RY6#1-transfected MIA-PaCa2 treated with the 4-PBA or DMSO. **(I-K)** Representative results of proliferation and apoptosis assays, including growth curves **(I)**, colony formation assays **(J)** and flow cytometric analysis of apoptosis rate **(K)** in vector or sh-P2RY6#1-transfected MIA-PaCa2 treated with 4-PBA or DMSO. Statistical analyses were derived from Unpaired t-test, error bars represent SD based on three independent experiments (n = 3), ^ns^ p >0.05, *p < 0.05, **p < 0.01, ***p < 0.001.

To further elucidate the mechanisms by which P2RY6 mediates the malignancy of PDAC cells, we performed RNA-seq on P2RY6-knockdown and vector-transfected MIA-PaCa2 to uncover associated transcriptional changes. The analysis identified 868 downregulated and 1,256 upregulated genes in P2RY6-knockdown MIA-PaCa2 (**Figures 9A, B**). GO analysis revealed that upregulated genes were significantly enriched in pathways related to antigen presentation, immune activation, ERS, and intrinsic apoptosis, whereas downregulated genes were enriched in pathways related to DNA replication (**Figures 9C, D**). To validate the transcriptomic alterations, we assess the mRNA level of selected genes associated with MHC antigen presentation and ERS pathway by RT-qPCR, which confirmed the RNA-seq findings (**Figure 9E**). Flow cytometry analysis further demonstrated that P2RY6 knockdown induced a significant G1-S phase arrest in MIA-PaCa2 (**Figure 9F**). Combined with the earlier EdU proliferation assay results, these findings indicate that P2RY6 knockdown markedly impairs the DNA replication capability of PDAC cells.

Notably, transcriptomic analysis revealed significant upregulation of ERS-related genes, such as DDIT3, HSPA5, and ERO1A, suggesting a pivotal role of ERS in the effects mediated by P2RY6 knockdown (**Figures 9A, C**). To verify this, WB analysis showed that P2RY6 knockdown significantly increased the expression of ERS markers (DDIT3 and downstream proteins such as TNFRSF10B, ERO1A, and PPP1R15A) and apoptosis-related proteins (**Figure 9G**). To assess the functional relevance of ERS, we treated P2RY6-knockdown MIA-PaCa2 with the ERS inhibitor 4-PBA, which markedly reduced ERS and apoptosis marker (**Figure 9H**). Subsequent CCK-8, colony formation, and flow cytometry further confirmed that 4-PBA partially reversed the impaired proliferation and increased apoptosis caused P2RY6 knockdown (**Figures 9I–K**). In summary, our findings demonstrate that P2RY6 knockdown suppresses PDAC cell proliferation and induces apoptosis primarily through ERS activation.

## 4 Discussion

PDAC is characterized by poor prognosis, resulting from challenges in early diagnosis, low surgical resection rates, limited drug efficacy, and high rates of postoperative recurrence and metastasis. Addressing the barriers to early diagnosis and precision therapy remains an urgent need. Recent studies have revealed that elevated expression of efferocytosis-related positive regulators promotes tumor cell survival, metastasis. In this study, we identified significant activation of the efferocytosis pathway in PDAC tissue and developed a prognostic model, EFFscore. The EFFscore demonstrated remarkable predictive performance and provided insights into the biological characteristics of tumor cells. Further investigation revealed that the essential mediator P2RY6 was significantly upregulated in PDAC tissues and cell lines. P2RY6 knockdown exerted anti-tumor effects by enhancing ERS and the MHC antigen presentation pathway. Moreover, the P2RY6 receptor antagonist MRS-2578 has been demonstrated as a promising therapeutic agent for PDAC. In conclusion, our study offers a novel perspective for the clinical assessment and targeted therapy of PDAC, providing a potential foundation for improving outcomes in this challenging malignancy.

### 4.1 Assessment of prognostic prediction and tumor biological characteristics in PDAC patients based on EFFscore

Although AJCC staging and serum CA19–9 level remain the most widely used prognostic tools in current clinical practice for PDAC, their predictive accuracy is suboptimal (44–46). For example, CA19–9 exhibits a sensitivity of only 70–80% and lacks reliability in Lewis antigen–negative individuals, while AJCC staging primarily reflects anatomical extent without capturing the underlying tumor biology (44). Previous studies have reported that the prognostic AUC of CA19–9 for overall survival ranges from approximately 0.61 to 0.71, depending on the disease stage and cutoff values. Similarly, the latest AJCC TNM staging system typically achieves AUCs below 0.70 when used alone (45, 47). In contrast, our EFFscore model integrates transcriptomic features related to efferocytosis, thereby capturing tumor-intrinsic biological characteristics. EFFscore consistently outperformed traditional metrics in survival stratification and remained an independent prognostic factor in multivariate analyses. These findings suggest that EFFscore may offer enhanced prognostic resolution beyond conventional staging systems and biomarkers. Nonetheless, its clinical utility still requires validation in larger and multicenter cohorts to ensure broader applicability.

The malignancy of PDAC has been attributed to its complex genome variation, which collectively orchestrate tumor initiation and progression (2). Accumulating evidence indicates that both the type and dosage of genome variation significantly impact patient prognosis. In particular, increased allele dosage of *KRAS* mutations has been recognized as a critical indicator of disease progression and an independent biomarker of poor prognosis (48). In our study, we observed that tumors with a high EFFscore exhibit a greater mutation burden, elevated CNV levels, and poorer differentiated. Consistently, single-cell analysis revealed a progressive increase in EFFscore from PanIN-like to highly proliferative subclusters, accompanied by transcriptional reprogramming related to genomic instability, metabolic reprogramming, and activation of oncogenic signaling. Furthermore, the predominant enrichment of EFFscore within tumor cells, rather than other microenvironmental components, supports its role in reflecting cell-intrinsic oncogenic programs. Collectively, these findings support EFFscore as a robust biomarker linking genome instability to phenotypic malignancy in PDAC, with promising potential for refining prognostic assessment and guiding personalized therapeutic strategies.

The intricate interplay among microenvironmental cells serves as a critical external driver of tumor progression. Cancer-associated fibroblasts (CAFs), which are highly heterogeneous and abundant in the PDAC microenvironment, play pivotal roles in promoting tumor proliferation, metastasis, drug resistance, and immune evasion (49). ANGPTL4, a pro-angiogenic factor involved in lipid metabolism, has been shown to promote fibroblast proliferation, migration, and collagen production (50–52). Similarly, MDK and PTN, members of the heparin-binding growth factor family, are extensively involved in tumor cell proliferation, EMT, angiogenesis, and immune suppression (53, 54). Previous studies have identified ANGPTL4 and MDK as key factors in inducing CAFs transformation and mediating renal fibrosis (55, 56). We hypothesize that the high EFFscore subgroup may secrete ANGPTL4 and MDK to activate myCAF populations, particularly *LRRC15*^+^ Fib and *STRA6*^+^ Fib, thereby driving late-stage ECM remodeling and promoting tumor invasion and metastasis. In contrast, the low EFFscore subgroup likely recruits and promotes fibroblast proliferation through the PDGFA-PDGFRA/B axis, facilitating the construction of an early-stage TME (57, 58). These findings highlight how EFFscore-defined tumor cell states differentially engage and reprogram CAFs, thereby dynamically reshaping the stromal architecture to support PDAC progression.

The limited efficacy of immunotherapy in PDAC is primarily attributed to its profoundly immunosuppressive microenvironment. Our findings revealed that the high EFFscore subgroup exhibits stronger MIF-CD74 signaling activity with immune cells. Numerous studies have demonstrated that the MIF-CD74 axis promotes tumor progression by activating oncogenic pathways and driving immune suppression, while its blockade can effectively restore antitumor immune responses within the TME (59–62). Similarly, enhanced activity of the MDK-NCL and ANXA1-FPR1 signaling axes may contribute to tumor immune evasion by promoting macrophage recruitment, driving polarization toward an immunosuppressive phenotype (63, 64). Conversely, the low EFFscore subgroup exhibits enhanced CXCL16-CXCR6 signaling, which may facilitate CD8^+^ T cell infiltration and enhance tumor-effector function, thereby supporting antitumor immunity (65–67). These findings provide mechanistic insights into the heterogeneous immune infiltration and varied immunotherapeutic responses observed in patients with distinct EFFscore subgroups.

The abundant infiltration of immunosuppressive myeloid cells, driven in part by efferocytosis, is a defining feature of the PDAC microenvironment (68). Our findings indicate that myeloid cells engage in extensive crosstalk with PDAC cells—particularly those in high EFFscore subgroup—through multiple signaling axes, including SPP1-CD44/(ITGAV+ITGB), OSM-(OSMR+IL6ST), and RETN-CAP1. Among these, SPP1, secreted by TAMs, functions as an adhesion molecule that interacts with integrins and CD44. This signaling cascade induces EMT, sustain cancer stemness, mediate chemoresistance and facilitate immune evasion, ultimately leading to poor prognosis (69–71). In our analysis, *C1QC*^+^ TAM and *SPP1*^+^ TAM emerged as the essential populations driving SPP1-mediated signaling, contributing to PDAC progression. Oncostatin M (OSM), an IL-6 family cytokine primarily secreted by immune cells, has been shown to activate the STAT3 pathway in tumor cells via the OSM receptor (OSMR), thereby promoting tumor proliferation and metastasis (72). Similarly, RETN can facilitate EMT and sustain cancer stemness through CAP1-dependent mechanisms (73). Collectively, the high EFFscore subgroup demonstrates intensified crosstalk with distinct microenvironmental components. This pattern reflects hallmarks of advanced-stage malignancy and highlighting the critical role of tumor-intrinsic features in orchestrating the PDAC microenvironment.

### 4.2 The oncogenic functions of P2RY6 in PDAC

The EFFscore comprises three genes—*ADAM9*, *P2RY6* and *CD36*—all of which have been closely associated with tumor progression. Among them, ADAM9, a zinc-dependent metalloproteinases widely overexpressed in various cancers. Its overexpression promotes tumor invasion and migration and correlates with poor prognosis (74–76). Recent studies have revealed that ADAM9 promotes tumor progression by protecting KRAS from lysosomal degradation and reducing its interaction with plasminogen activator inhibitor-1 (PAI-1) and microtubule-associated protein 1 light chain 3 (LC3) (77). The anti-ADAM9 antibody-drug conjugate (ADC) IMGC936 has demonstrated potent antitumor activity in ADAM9-positive tumor cells and xenograft models. It is currently undergoing clinical trials for advanced solid tumors (NCT04622774) (78). CD36, a scavenger receptor expressed in various cell types, mediates lipid uptake, immune recognition, inflammation and molecular adhesion (79). Research has shown that CD36 expression is significantly reduced in pancreatic cancer cell lines and tumor tissues, which may enhance tumor cell motility by weakening ECM adhesion and collagen-binding capacity, thereby promoting metastasis and poor prognosis (79, 80). Notably, after neoadjuvant therapy, PDAC cells upregulate CD36 to facilitate metabolic reprogramming under chemotherapy-induced stress, and its targeting has been shown to enhance therapeutic efficacy (81).

Among the genes comprising the EFFscore, P2RY6 emerged as the top-weighted feature gene, exhibiting consistent upregulation in PDAC cells and tissues. As a GPCR activated by extracellular UDP, P2RY6 has been implicated in diverse tumor-promoting processes. Notably, UDP accumulated in the PDAC microenvironment under environmental stress serves as an alternative to glucose, potentially driving sustained P2RY6 activation (82). In our study, elevated P2RY6 expression was associated with higher tumor grade and worse prognosis, consistent with EFFscore-based stratification. To explore the downstream effects of P2RY6, we performed transcriptomic profiling following P2RY6 knockdown in PDAC cells. The results revealed extensive transcriptional reprogramming, with upregulated genes significantly enriched in immune activation and ERS-related apoptotic signaling pathway. These findings suggest that P2RY6 may promote tumor progression through both cell-intrinsic mechanisms and microenvironmental modulation.

ERS is a critical signaling pathway activated in response to misfolded proteins, protein aggregation, and calcium homeostasis disruption (83). Specific intracellular gene expression changes and extrinsic stress can both induce ERS, which subsequently activates the UPR to preserve protein homeostasis and support tumor cell survival (83). However, when ER stress exceeds the threshold of tolerance or the adaptive UPR fails to restore protein homeostasis, imbalanced ERS triggers a terminal UPR response that leads to cell cycle arrest, mitochondrial dysfunction, ultimately resulting in apoptosis (83). In our study, P2RY6 knockdown significantly increased ERS level in PDAC cells, which is consistent with the enrichment of UPR pathways identified in the Low EFFscore subgroup via GSVA analysis. Further analysis demonstrated that the ERS inhibitor 4-PBA partially alleviated the proliferation inhibition and apoptosis induced by P2RY6 knockdown. This finding confirms the critical role of P2RY6-mediated ERS dysregulation in modulating the biological function of PDAC. Notably, recent studies have shown that ERS-induced immunogenic cell death can reshape macrophage behavior in the TME (84). Ferroptosis can promote M1-like polarization, while necroptosis can enhance metastatic niche formation through macrophage extracellular trap (MET) activation (85, 86). Consistently, our co-culture experiments revealed that P2RY6 knockdown exhibited a shift in macrophage polarization indicative of immune activation. These results suggest that P2RY6-mediated ERS may influence tumor progression not only through intrinsic apoptotic signaling, but also possibly by modulating the tumor immune microenvironment through its regulation of macrophage function.

Importantly, we found that inhibition of ERS did not fully restore the biological function of PDAC, suggesting that P2RY6 may promote tumor progression through additional mechanisms. As a Gq protein-coupled receptor, P2RY6 has been reported to activate multiple oncogenic signaling pathways, including MAPK, NF-κB, PI3K/Akt and Rho/ROCK, which contribute to tumor cell proliferation, migration, survival, and invasion (21). For instance, P2RY6 has been shown to promote skin cancer development by modulating MAPK/ERK1-mediated Hippo/YAP and Wnt/β-catenin signaling, and to enhance migration in lung and colon cancer via the Rho/ROCK pathway (87, 88). In addition, P2RY6 protects colon cancer cells from TNF-α-induced apoptosis by activating AKT-mediated phosphorylation of the X-linked inhibitor of apoptosis protein (XIAP) (89). Beyond these tumor-intrinsic oncogenic effects, emerging evidence suggests that P2RY6 may also facilitate immune evasion. Tumor-intrinsic P2RY6 has been shown to activate the GNAQ/GNA11–PLCB pathway. This activation upregulates Ptgs1 and Ptgs2, the rate-limiting enzymes for prostaglandin synthesis, thereby increasing PGE2 production and suppressing anti-tumor immunity (22). Collectively, these findings suggest that additional studies are warranted to delineate the complex mechanisms by which tumor-intrinsic P2RY6 contributes to PDAC progression.

### 4.3 Challenges and considerations for targeting P2RY6 in PDAC

While these findings highlight the therapeutic potential of targeting P2RY6—particularly through pharmacological antagonists such as MRS-2578—it remains critical to consider the functional complexity of P2RY6 signaling. First, although P2RY6 is widely recognized as a pro-inflammatory mediator in various inflammatory diseases, the immunological landscape of PDAC is fundamentally distinct. The PDAC microenvironment is profoundly immunosuppressive, characterized by dysfunctional and exhausted immune cells. Emerging evidence indicates that PDAC cells promote the infiltration and immunosuppressive activation of *P2RY6*^+^ macrophages (20), suggesting that both tumor and immune cells within the PDAC microenvironment may exploit P2RY6 signaling to facilitate tumor progression. Thus, these findings imply that targeting P2RY6 with systemic MRS-2578 is unlikely to exacerbate immunosuppression in PDAC. Second, both our findings and previous studies underscore the critical role of P2RY6 in myeloid and tumor cells (**Supplementary Table S3**); however, its function in other tumor microenvironmental components remains largely unexplored. Notably, P2RY6 is also highly expressed in endothelial cells, suggesting that its potential involvement in vascular remodeling and immune regulation warrants further investigation. Third, current evidence regarding MRS-2578 is derived primarily from immunodeficient or simplified preclinical models. To better evaluate its therapeutic utility, systemic effects of MRS-2578 need to be validated in immunocompetent models that more comprehensively represent the change of PDAC microenvironment. Furthermore, comprehensive toxicological assessments and well-designed clinical studies are essential to evaluate its safety profile, off-target effects, bioavailability and overall translational potential.

In addition, some limitations of the present study should be acknowledged. First, the clinical validation cohort and functional experiments were conducted with relatively small sample sizes, which may limit the generalizability and statistical robustness of the findings. Second, the use of retrospective public datasets introduces inherent heterogeneity due to variations in sample collection, processing methods, and clinical annotation, which may compromise the consistency and reliability of the results. To strengthen the clinical applicability of the EFFscore and P2RY6-targeted strategies, future studies should include large-scale, prospective, multicenter cohorts for external validation. Furthermore, the evidence and mechanistic basis for the proposed tumor-suppressive role of P2RY6 by ERS and immunoregulation remain limited, necessitating more well-controlled experiments to substantiate these findings.

## 5 Conclusion

We developed an effective prognostic model, EFFscore, which demonstrates robust predictive performance and accurately reflects the malignant potential of PDAC. Subsequent analysis identified P2RY6 as a key effector gene within the model, exhibiting significant upregulation in PDAC tissues and cell lines. Functional inhibition of P2RY6 significantly suppressed the malignant phenotype of PDAC cells by enhancing immune activation and promoting ERS–related apoptotic signaling. In summary, this study introduces a novel efferocytosis-related prognostic model for PDAC and highlights P2RY6 as a promising therapeutic target. Future investigations should prioritize clinical validation and explore the potential synergy between P2RY6 inhibition and immunotherapy to advance personalized treatment strategies.

## Data Availability

The datasets presented in this study can be found in online repositories. The names of the repository/repositories and accession number(s) can be found in the article/**Supplementary Material**.

## Supplementary material

Supplementary Figure 1 Heatmap of DE-ERGs and Optimization of the Prognostic Model. **(A)** Venn diagram depicts the intersection of the DEGs between tumor and normal tissue (n = 5871) and the efferocytosis-related gene set (n = 158). **(B)** Heatmap plot shows the expression of DE-ERGs in normal tissue (GTEx dataset, blue) and PDAC tissue (TCGA-PAAD dataset, red). **(C)** Adjustment path plot from the LASSO regression model, identifies the optimal λ value through ten-fold cross-validation. **(D)** Results of the LASSO-Cox analysis for the twelve most prognostically significant DE-ERGs.

Supplementary Figure 2 Construction and Evaluation of a Nomogram for Predicting the Survival in PDAC Patients from the TCGA Cohort. **(A, B)** Forest plots of univariate **(A)** and multivariate **(B)** Cox regression analysis of PDAC patients based on EFFscore and clinical characteristics, including age, sex, grade, and stage. **(C)** The prognostic nomogram model was constructed using EFFscore and clinical indicators (age, sex, grade, and stage) to predict 1-year, 3-year, and 5-year OS in PDAC patients. **(D-F)** Calibration curves assess the performance of the nomogram in predicting 1-year **(D)**, 3-year **(E)**, and 5-year **(F)** OS. The gray diagonal line represents the ideal prediction, while the blue line indicates the observed prediction. The closer the blue line is to the gray line, the higher the accuracy of the model in predicting survival probabilities. **(G)** ROC curve for the nomogram demonstrates its predictive performance.

Supplementary Figure 3 Genomic Variation Level in High- and Low-EFFscore Patient Groups. **(A, B)** Genome mutation profile analysis: Comparative analysis of mutation characteristics in High-EFFscore **(A)** and Low-EFFscore **(B)** patients, including mutation classification, mutation type, SNV class, variants per sample, distribution of mutation classifications, and frequently mutated genes. **(C, D)** Somatic mutation waterfall plot for the High-EFFscore patients **(C)** and Low-EFFscore patients **(D)**. Each column represents an individual patient, with the top bar plot indicates TMB. Numbers on the right denote the mutation frequency for each gene, and the adjacent bar chart shows the proportion of each variant type. **(E)** Comparison of total TMB level between High- and Low-EFFscore patients. **(F)** K-M survival curve shows the survival of PDAC patients with high and low TMB based on the median TMB value. **(G–J)** CNV frequencies of *ADAM9*, *P2RY6*, and *CD36* in the High-EFFscore group **(G)** and Low-EFFscore group **(H)**. Statistical analysis was derived from unpaired t-test, * p < 0.05.

Supplementary Figure 4 Comparison of Drug Sensitivity Between High- and Low-EFFscore Patients. Difference in tumor drug sensitivity between High-and Low-EFFscore patient groups. **(A-L)** Evaluation of drug sensitivity in High- and Low-EFFscore patients for chemotherapeutic drugs:Gemcitabine **(A)**, Fluorouracil **(B)**, Oxaliplatin **(C)**, Irinotecan **(D)**, Carboplatin **(E)**; tyrosine kinase inhibitors: Sorafenib **(F)**, Sunitinib **(G)**, Canertinib **(H)**, Masitinib **(I)**; JAK inhibitor: Ruxolitinib **(J)**; NOTCH inhibitor: MK-0752 **(K)**; TGF-β inhibitor: LY-2157299 **(L)**. Statistical analyses were derived from Mann-Whitney U test, *p < 0.05, **p < 0.01, ***p < 0.001.

Supplementary Figure 5 Dimensionality Reduction, Clustering and CNV Analysis of Ductal Cells in the GSE194247 Dataset. **(A)** UMAP plot depicts the clustering of all cells into distinct clusters after dimensionality reduction. **(B, C)** Bubble plot shows the expression of marker genes across major clusters **(B)** and PDAC subclusters **(C)**. Bubble size represents the proportion of cells expressing marker genes, while color indicates the average expression of the genes within the cells. **(D)** CNV analysis of two types of ductal epithelial cell using inferCNV, with all non-ductal cells serving as the reference control. **(E)** Violin plot shows the CNV level across PDAC subclusters. **(F)** The difference of CNV between High- and Low-EFFscore subgroup. Statistical analysis was derived from unpaired t-test, ***p < 0.001.

Supplementary Figure 6 Dimensionality Reduction, Clustering and EFFscore Distribution in the CRA001160 Dataset. **(A, B)** UMAP plots depicts the distribution of cells based on sample origin **(A)** and the distinct clusters following dimensionality reduction **(B)**. **(C)** Bubble plot shows the expression profiles of marker genes across clusters. Bubble size represents the proportion of cells expressing marker genes, while color indicates the average expression of the genes within the cells. **(D)** Heatmap plot shows the Expression and distribution of *ADAM9*, *P2RY6*, *CD36*, and EFFscore across different clusters.

Supplementary Figure 7 Analysis of the Intercellular Communication Network in TME and Ligand-Receptor Interaction Between Myeloid Cell and PDAC cell. **(A)** CellChat analysis of intercellular communication in TME depicts interaction strength **(A)** and frequency **(B)** among various clusters. Node size represents the size of each cluster, while the thickness of the connecting lines indicates the strength and number of interactions. **(C)** Ligand-receptor interactions between myeloid cells (donors) and distinct EFFscore subgroups (recipient). Dot color represents interaction probability, and dot size indicates the statistical significance (p-value).

Supplementary Figure 8 Expression of Marker Genes in Fibroblast, Myeloid, and T Cell Subclusters. **(A–C)** Bubble plot showing the expression patterns of marker genes within fibroblast **(A)**, myeloid cell **(B)**, T cell **(C)** subclusters. Bubble size represents the proportion of cells expressing marker genes, while color indicates the average expression of the genes within the cells, SP: Single positive.

Supplementary Figure 9 P2RY6 Promotes Immune Evasion and Shapes the Immunosuppressive Microenvironment in PDAC. **(A, B)** TIDE analysis evaluates immunotherapy response, including TIDE score, exclusion score, dysfunction score **(A)** and immunotherapy response **(B)**, with red indicating responders and blue indicating non-responders. **(C)** RT-qPCR analysis of markers for M1-like pro-inflammatory and M2-like anti-inflammatory phenotypes after co-culture with MIA-PaCa2 and Capan-1 cells. Statistical analyses include Mann-Whitney U test **(A)** and Chi-square test **(B)** and Unpaired t-test **(C)**, error bars represent SD based on three independent experiments (n = 3), ** p < 0.01, *** p < 0.001.

## Glossary

PDAC: Pancreatic ductal adenocarcinoma
OS: Overall survival
PanIN: Pancreatic intraepithelial neoplasia
IPMN: Intraductal papillary mucinous neoplasm
TME: Tumor microenvironment, ECM, Extracellular matrix
scRNA-seq: Single-cell RNA sequence
TCGA: The Cancer Genome Atlas
GTEx: Genotype-Tissue Expression Project
KEGG: Kyoto Encyclopedia of Genes and Genomes
GEO: Gene Expression Omnibus
FC: Fold change
DEGs: Differentially expressed genes
DE-ERGs: Differentially expressed efferocytosis-related genes
GO: Gene Ontology
LASSO: Least absolute shrinkage and selection operator
ROC: Receiver operating characteristic
K-M: Kaplan-Meier
INS: insertion mutations
DEL: deletion mutations
CNV: Copy number variation
TMB: Tumor mutation burden
IC50: Half maximal inhibitory concentration
CTRP: Cancer Therapeutics Response Portal
PCA: Principal component analysis
UMAP: Uniform Manifold Approximation and Projection
MC: Malignant cell
GSVA: Gene set variation analysis
MSigDB: Molecular Signatures Database
IHC: Immunohistochemistry
IRS: Immunoreactive score
PI: Propidium
GSEA: Gene set enrichment analysis
SNPs: Single nucleotide polymorphisms
ESMO: European Society for Medical Oncology
EMT: Epithelial-mesenchymal transition
UPR: Unfolded protein response
BP: Biological Process
CC: Cellular Component
MF: Molecular Function
ERS: Endoplasmic reticulum stress
RT-qPCR: Reverse Transcription Quantitative Polymerase Chain Reaction
WB: Western blot

## References

[1] Siegel RL Giaquinto AN Jemal A . Cancer statistics, 2024. CA Cancer J Clin. (2024) 74:12–49. doi: 10.3322/caac.21820, PMID: 38230766

[2] Halbrook CJ Lyssiotis CA Pasca di Magliano M Maitra A . Pancreatic cancer: Advances and challenges. Cell. (2023) 186:1729–54. doi: 10.1016/j.cell.2023.02.014, PMID: 37059070 PMC10182830

[3] Wood LD Canto MI Jaffee EM Simeone DM . Pancreatic cancer: pathogenesis, screening, diagnosis, and treatment. Gastroenterology. (2022) 163:386–402.e1. doi: 10.1053/j.gastro.2022.03.056, PMID: 35398344 PMC9516440

[4] Stoop TF Javed AA Oba A Koerkamp BG Seufferlein T Wilmink JW . Pancreatic cancer. Lancet. (2025) 405:1182–202. doi: 10.1016/S0140-6736(25)00261-2, PMID: 40187844

[5] Yu B Shao S Ma W . Frontiers in pancreatic cancer on biomarkers, microenvironment, and immunotherapy. Cancer Lett. (2025) 610:217350. doi: 10.1016/j.canlet.2024.217350, PMID: 39581219

[6] Bear AS Vonderheide RH O’Hara MH . Challenges and opportunities for pancreatic cancer immunotherapy. Cancer Cell. (2020) 38:788–802. doi: 10.1016/j.ccell.2020.08.004, PMID: 32946773 PMC7738380

[7] Yamamoto K Venida A Yano J Biancur DE Kakiuchi M Gupta S . Autophagy promotes immune evasion of pancreatic cancer by degrading MHC-I. Nature. (2020) 581:100–5. doi: 10.1038/s41586-020-2229-5, PMID: 32376951 PMC7296553

[8] Casey SC Tong L Li Y Do R Walz S Fitzgerald KN . MYC regulates the anti-tumor immune response through CD47 and PD-L1. Science. (2016) 352:227–31. doi: 10.1126/science.aac9935, PMID: 26966191 PMC4940030

[9] Zhou Y Yao Y Deng Y Shao A . Regulation of efferocytosis as a novel cancer therapy. Cell Commun Signal. (2020) 18:71. doi: 10.1186/s12964-020-00542-9, PMID: 32370748 PMC7199874

[10] Doran AC Yurdagul A Tabas I . Efferocytosis in health and disease. Nat Rev Immunol. (2020) 20:254–67. doi: 10.1038/s41577-019-0240-6, PMID: 31822793 PMC7667664

[11] Liu S Zhang H Li Y Zhang Y Bian Y Zeng Y . S100A4 enhances protumor macrophage polarization by control of PPAR-γ-dependent induction of fatty acid oxidation. J Immunother Cancer. (2021) 9:e002548. doi: 10.1136/jitc-2021-002548, PMID: 34145030 PMC8215236

[12] Hou Z Lu F Lin J Wu Y Chen L Fang H . Loss of Annexin A1 in macrophages restrains efferocytosis and remodels immune microenvironment in pancreatic cancer by activating the cGAS/STING pathway. J Immunother Cancer. (2024) 12:e009318. doi: 10.1136/jitc-2024-009318, PMID: 39237260 PMC11381726

[13] Astuti Y Raymant M Quaranta V Clarke K Abudula M Smith O . Efferocytosis reprograms the tumor microenvironment to promote pancreatic cancer liver metastasis. Nat Cancer. (2024) 5:774–90. doi: 10.1038/s43018-024-00731-2, PMID: 38355776 PMC11136665

[14] Di Carlo SE Raffenne J Varet H Ode A Granados DC Stein M . Depletion of slow-cycling PDGFRα+ADAM12+ mesenchymal cells promotes antitumor immunity by restricting macrophage efferocytosis. Nat Immunol. (2023) 24:1867–78. doi: 10.1038/s41590-023-01642-7, PMID: 37798557 PMC10602852

[15] Qiu H Shao Z Wen X Liu Z Chen Z Qu D . Efferocytosis: An accomplice of cancer immune escape. BioMed Pharmacother. (2023) 167:115540. doi: 10.1016/j.biopha.2023.115540, PMID: 37741255

[16] Novak I Yu H Magni L Deshar G . Purinergic signaling in pancreas—From physiology to therapeutic strategies in pancreatic cancer. Int J Mol Sci. (2020) 21:8781. doi: 10.3390/ijms21228781, PMID: 33233631 PMC7699721

[17] Anwar S Pons V Rivest S . Microglia purinoceptor P2Y6: an emerging therapeutic target in CNS diseases. Cells. (2020) 9:1595. doi: 10.3390/cells9071595, PMID: 32630251 PMC7407337

[18] Zhu Y Zhou M Cheng X Wang H Li Y Guo Y . Discovery of selective P2Y6R antagonists with high affinity and *in vivo* efficacy for inflammatory disease therapy. J Med Chem. (2023) 66:6315–32. doi: 10.1021/acs.jmedchem.3c00210, PMID: 37078976

[19] Li Y Zhou M Li H Dai C Yin L Liu C . Macrophage P2Y6 receptor deletion attenuates atherosclerosis by limiting foam cell formation through phospholipase Cβ/store-operated calcium entry/calreticulin/scavenger receptor A pathways. Eur Heart J. (2024) 45:268–83. doi: 10.1093/eurheartj/ehad796, PMID: 38036416

[20] Scolaro T Manco M Pecqueux M Amorim R Trotta R Van Acker HH . Nucleotide metabolism in cancer cells fuels a UDP-driven macrophage cross-talk, promoting immunosuppression and immunotherapy resistance. Nat Cancer. (2024) 5:1206–26. doi: 10.1038/s43018-024-00771-8, PMID: 38844817 PMC11358017

[21] Woods LT Forti KM Shanbhag VC Camden JM Weisman GA . P2Y receptors for extracellular nucleotides: contributions to cancer progression and therapeutic implications. Biochem Pharmacol. (2021) 187:114406. doi: 10.1016/j.bcp.2021.114406, PMID: 33412103 PMC8096679

[22] Xu X Lu Y Cao L Miao Y Li Y Cui Y . Tumor-intrinsic P2RY6 drives immunosuppression by enhancing PGE2 production. Cell Rep. (2024) 43(7):114469. doi: 10.1016/j.celrep.2024.114469, PMID: 38996067

[23] Yang S He P Wang J Schetter A Tang W Funamizu N . A novel MIF signaling pathway drives the Malignant character of pancreatic cancer by targeting NR3C2. Cancer Res. (2016) 76:3838–50. doi: 10.1158/0008-5472.CAN-15-2841, PMID: 27197190 PMC4930741

[24] Yang S Tang W Azizian A Gaedcke J Ströbel P Wang L . Dysregulation of HNF1B/Clusterin axis enhances disease progression in a highly aggressive subset of pancreatic cancer patients. Carcinogenesis. (2022) 43:1198–210. doi: 10.1093/carcin/bgac092, PMID: 36426859 PMC10122429

[25] Xiang G Dong X Xu T Feng Y He Z Ke C . A nomogram for prediction of postoperative pneumonia risk in elderly hip fracture patients. Risk Manag Healthc Policy. (2020) 13:1603–11. doi: 10.2147/RMHP.S270326, PMID: 32982518 PMC7502327

[26] Mayakonda A Lin D Assenov Y Plass C Koeffler H . Maftools: efficient and comprehensive analysis of somatic variants in cancer. Genome Res. (2018) 28(11):1747–56. doi: 10.1101/gr.239244.118, PMID: 30341162 PMC6211645

[27] Yoshihara K Shahmoradgoli M Martínez E Vegesna R Kim H Torres-Garcia W . Inferring tumour purity and stromal and immune cell admixture from expression data. Nat Commun. (2013) 4:2612. doi: 10.1038/ncomms3612, PMID: 24113773 PMC3826632

[28] Jiang P Gu S Pan D Fu J Sahu A Hu X . Signatures of T cell dysfunction and exclusion predict cancer immunotherapy response. Nat Med. (2018) 24:1550–8. doi: 10.1038/s41591-018-0136-1, PMID: 30127393 PMC6487502

[29] Maeser D Gruener RF Huang RS . oncoPredict: an R package for predicting *in vivo* or cancer patient drug response and biomarkers from cell line screening data. Brief Bioinform. (2021) 22:bbab260. doi: 10.1093/bib/bbab260, PMID: 34260682 PMC8574972

[30] Kim S Leem G Choi J Koh Y Lee S Nam S-H . Integrative analysis of spatial and single-cell transcriptome data from human pancreatic cancer reveals an intermediate cancer cell population associated with poor prognosis. Genome Med. (2024) 16:20. doi: 10.1186/s13073-024-01287-7, PMID: 38297291 PMC10832111

[31] Peng J Sun B-F Chen C-Y Zhou J-Y Chen Y-S Chen H . Single-cell RNA-seq highlights intra-tumoral heterogeneity and Malignant progression in pancreatic ductal adenocarcinoma. Cell Res. (2019) 29:725–38. doi: 10.1038/s41422-019-0195-y, PMID: 31273297 PMC6796938

[32] Hao Y Hao S Andersen-Nissen E Mauck WM Zheng S Butler A . Integrated analysis of multimodal single-cell data. Cell. (2021) 184:3573–3587.e29. doi: 10.1016/j.cell.2021.04.048, PMID: 34062119 PMC8238499

[33] McGinnis CS Murrow LM Gartner ZJ . DoubletFinder: doublet detection in single-cell RNA sequencing data using artificial nearest neighbors. Cell Syst. (2019) 8:329–337.e4. doi: 10.1016/j.cels.2019.03.003, PMID: 30954475 PMC6853612

[34] Korsunsky I Millard N Fan J Slowikowski K Zhang F Wei K . Fast, sensitive and accurate integration of single-cell data with Harmony. Nat Methods. (2019) 16:1289–96. doi: 10.1038/s41592-019-0619-0, PMID: 31740819 PMC6884693

[35] Patel AP Tirosh I Trombetta JJ Shalek AK Gillespie SM Wakimoto H . Single-cell RNA-seq highlights intratumoral heterogeneity in primary glioblastoma. Science. (2014) 344:1396–401. doi: 10.1126/science.1254257, PMID: 24925914 PMC4123637

[36] Cao J Spielmann M Qiu X Huang X Ibrahim DM Hill AJ . The single-cell transcriptional landscape of mammalian organogenesis. Nature. (2019) 566:496–502. doi: 10.1038/s41586-019-0969-x, PMID: 30787437 PMC6434952

[37] Wu T Hu E Xu S Chen M Guo P Dai Z . clusterProfiler 4.0: A universal enrichment tool for interpreting omics data. Innovation (Camb). (2021) 2:100141. doi: 10.1016/j.xinn.2021.100141, PMID: 34557778 PMC8454663

[38] Jin S Guerrero-Juarez CF Zhang L Chang I Ramos R Kuan C-H . Inference and analysis of cell-cell communication using CellChat. Nat Commun. (2021) 12:1088. doi: 10.1038/s41467-021-21246-9, PMID: 33597522 PMC7889871

[39] Xu S Zhan M Jiang C He M Yang L Shen H . Genome-wide CRISPR screen identifies ELP5 as a determinant of gemcitabine sensitivity in gallbladder cancer. Nat Commun. (2019) 10:5492. doi: 10.1038/s41467-019-13420-x, PMID: 31792210 PMC6889377

[40] Zhang S Lin H Wang J Rui J Wang T Cai Z . Sensing ceramides by CYSLTR2 and P2RY6 to aggravate atherosclerosis. Nature. (2025) 641:476–85. doi: 10.1038/s41586-025-08792-8, PMID: 40049228

[41] Vasan N Baselga J . Hyman DM. A view on drug resistance in cancer. Nature. (2019) 575:299–309. doi: 10.1038/s41586-019-1730-1, PMID: 31723286 PMC8008476

[42] Conroy T Pfeiffer P Vilgrain V Lamarca A Seufferlein T O’Reilly EM . Pancreatic cancer: ESMO Clinical Practice Guideline for diagnosis, treatment and follow-up. Ann Oncol. (2023) 34:987–1002. doi: 10.1016/j.annonc.2023.08.009, PMID: 37678671

[43] Cui Zhou D Jayasinghe RG Chen S Herndon JM Iglesia MD Navale P . Spatially restricted drivers and transitional cell populations cooperate with the microenvironment in untreated and chemo-resistant pancreatic cancer. Nat Genet. (2022) 54:1390–405. doi: 10.1038/s41588-022-01157-1, PMID: 35995947 PMC9470535

[44] Luo G Jin K Deng S Cheng H Fan Z Gong Y . Roles of CA19–9 in pancreatic cancer: Biomarker, predictor and promoter. Biochim Biophys Acta (BBA) - Rev Cancer. (2021) 1875:188409. doi: 10.1016/j.bbcan.2020.188409, PMID: 32827580

[45] Van Roessel S Kasumova GG Verheij J Najarian RM Maggino L De Pastena M . International validation of the eighth edition of the american joint committee on cancer (AJCC) TNM staging system in patients with resected pancreatic cancer. JAMA Surg. (2018) 153:e183617. doi: 10.1001/jamasurg.2018.3617, PMID: 30285076 PMC6583013

[46] Vernerey D Huguet F Vienot A Goldstein D Paget-Bailly S Van Laethem J-L . Prognostic nomogram and score to predict overall survival in locally advanced untreated pancreatic cancer (PROLAP). Br J Cancer. (2016) 115:281–9. doi: 10.1038/bjc.2016.212, PMID: 27404456 PMC4973163

[47] Dong Q Yang X Zhang Y Jing W Zheng L Liu Y . Elevated serum CA19–9 level is a promising predictor for poor prognosis in patients with resectable pancreatic ductal adenocarcinoma: a pilot study. World J Surg Oncol. (2014) 12:171. doi: 10.1186/1477-7819-12-171, PMID: 24890327 PMC4064278

[48] Varghese AM Perry MA Chou JF Nandakumar S Muldoon D Erakky A . Clinicogenomic landscape of pancreatic adenocarcinoma identifies KRAS mutant dosage as prognostic of overall survival. Nat Med. (2025) 31(2):466–77. doi: 10.1038/s41591-024-03362-3, PMID: 39753968 PMC11835752

[49] Chhabra Y Weeraratna AT . Fibroblasts in cancer: Unity in heterogeneity. Cell. (2023) 186:1580–609. doi: 10.1016/j.cell.2023.03.016, PMID: 37059066 PMC11422789

[50] Park MS Kim SE Lee P Lee J-H Jung KH Hong S-S . Potential role of ANGPTL4 in cancer progression, metastasis, and metabolism: a brief review. BMB Rep. (2024) 57:343–51. doi: 10.5483/BMBRep.2024-0082, PMID: 39044455 PMC11362140

[51] Jamil S Mousavizadeh R Roshan-Moniri M Tebbutt SJ Mccormack RG Duronio V . Angiopoietin-like 4 enhances the proliferation and migration of tendon fibroblasts. Med Sci Sports Exercise. (2017) 49:1769. doi: 10.1249/MSS.0000000000001294, PMID: 28398948

[52] Zhu X Zhang X Gu W Zhao H Hao S Ning Z . ANGPTL4 suppresses the profibrogenic functions of atrial fibroblasts induced by angiotensin II by up-regulating PPARγ. Iran J Basic Med Sci. (2023) 26:587–93. doi: 10.22038/IJBMS.2023.69196.15077, PMID: 37051105 PMC10083826

[53] Filippou PS Karagiannis GS Constantinidou A . Midkine (MDK) growth factor: a key player in cancer progression and a promising therapeutic target. Oncogene. (2020) 39:2040–54. doi: 10.1038/s41388-019-1124-8, PMID: 31801970

[54] Cai YQ Lv Y Mo ZC Lei J Zhu JL Zhong QQ . Multiple pathophysiological roles of midkine in human disease. Cytokine. (2020) 135:155242. doi: 10.1016/j.cyto.2020.155242, PMID: 32799009

[55] Srivastava SP Zhou H Shenoi R Morris M Lainez-Mas B Goedeke L . Renal Angptl4 is a key fibrogenic molecule in progressive diabetic kidney disease. Sci Adv. (2024) 10(49):eadn6068. doi: 10.1126/sciadv.adn6068, PMID: 39630889 PMC11616692

[56] Xu C Chen J Liang L Chen S Niu X Sang R . Midkine promotes renal fibrosis by stabilizing C/EBPβ to facilitate endothelial-mesenchymal transition. Commun Biol. (2024) 7:544. doi: 10.1038/s42003-024-06154-0, PMID: 38714800 PMC11076470

[57] Chu X Li X Zhang Y Dang G Miao Y Xu W . Integrative single-cell analysis of human colorectal cancer reveals patient stratification with distinct immune evasion mechanisms. Nat Cancer. (2024) 5:1409–26. doi: 10.1038/s43018-024-00807-z, PMID: 39147986

[58] Chen S-Y Lin J-S Lin H-C Shan Y-S Cheng Y-J Yang B-C . Dependence of fibroblast infiltration in tumor stroma on type IV collagen-initiated integrin signal through induction of platelet-derived growth factor. Biochim Biophys Acta. (2015) 1853:929–39. doi: 10.1016/j.bbamcr.2015.02.004, PMID: 25686533

[59] Bozzi F Mogavero A Varinelli L Belfiore A Manenti G Caccia C . MIF/CD74 axis is a target for novel therapies in colon carcinomatosis. J Exp Clin Cancer Res. (2017) 36:16. doi: 10.1186/s13046-016-0475-z, PMID: 28114961 PMC5260021

[60] Ghoochani A Schwarz MA Yakubov E Engelhorn T Doerfler A Buchfelder M . MIF-CD74 signaling impedes microglial M1 polarization and facilitates brain tumorigenesis. Oncogene. (2016) 35:6246–61. doi: 10.1038/onc.2016.160, PMID: 27157615

[61] De Azevedo RA Shoshan E Whang S Markel G Jaiswal AR Liu A . MIF inhibition as a strategy for overcoming resistance to immune checkpoint blockade therapy in melanoma. Oncoimmunology. (2020) 9:1846915. doi: 10.1080/2162402X.2020.1846915, PMID: 33344042 PMC7733907

[62] Liu L Wang J Wang Y Chen L Peng L Bin Y . Blocking the MIF-CD74 axis augments radiotherapy efficacy for brain metastasis in NSCLC via synergistically promoting microglia M1 polarization. J Exp Clin Cancer Res. (2024) 43:128. doi: 10.1186/s13046-024-03024-9, PMID: 38685050 PMC11059744

[63] Zhang S Zhang Y Song X Wang X Quan L Xu P . Immune escape between endoplasmic reticulum stress-related cancer cells and exhausted CD8+T cells leads to neoadjuvant chemotherapy resistance in ovarian cancer. Biochem Biophys Res Commun. (2024) 733:150686. doi: 10.1016/j.bbrc.2024.150686, PMID: 39278093

[64] Zheng Y Jiang H Yang N Shen S Huang D Jia L . Glioma-derived ANXA1 suppresses the immune response to TLR3 ligands by promoting an anti-inflammatory tumor microenvironment. Cell Mol Immunol. (2024) 21:47–59. doi: 10.1038/s41423-023-01110-0, PMID: 38049523 PMC10757715

[65] Di Pilato M Kfuri-Rubens R Pruessmann JN Ozga AJ Messemaker M Cadilha BL . CXCR6 positions cytotoxic T cells to receive critical survival signals in the tumor microenvironment. Cell. (2021) 184:4512–4530.e22. doi: 10.1016/j.cell.2021.07.015, PMID: 34343496 PMC8719451

[66] Lesch S Blumenberg V Stoiber S Gottschlich A Ogonek J Cadilha BL . T cells armed with C-X-C chemokine receptor type 6 enhance adoptive cell therapy for pancreatic tumours. Nat BioMed Eng. (2021) 5:1246–60. doi: 10.1038/s41551-021-00737-6, PMID: 34083764 PMC7611996

[67] Su W Saravia J Risch I Rankin S Guy C Chapman NM . CXCR6 orchestrates brain CD8+ T cell residency and limits mouse Alzheimer’s disease pathology. Nat Immunol. (2023) 24:1735–47. doi: 10.1038/s41590-023-01604-z, PMID: 37679549 PMC11102766

[68] Xiong J Wang H Wang Q . Suppressive myeloid cells shape the tumor immune microenvironment. Adv Biol (Weinh). (2021) 5:e1900311. doi: 10.1002/adbi.201900311, PMID: 33729699

[69] Kumari A Kashyap D Garg VK . Osteopontin in cancer. Adv Clin Chem. (2024) 118:87–110. doi: 10.1016/bs.acc.2023.11.002, PMID: 38280808

[70] Nallasamy P Nimmakayala RK Karmakar S Leon F Seshacharyulu P Lakshmanan I . Pancreatic tumor microenvironment factor promotes cancer stemness via SPP1-CD44 axis. Gastroenterology. (2021) 161:1998–2013.e7. doi: 10.1053/j.gastro.2021.08.023, PMID: 34418441 PMC10069715

[71] Briones-Orta MA Avendaño-Vázquez SE Aparicio-Bautista DI Coombes JD Weber GF Syn W-K . Osteopontin splice variants and polymorphisms in cancer progression and prognosis. Biochim Biophys Acta Rev Cancer. (2017) 1868:93–108.A. doi: 10.1016/j.bbcan.2017.02.005, PMID: 28254527

[72] Geethadevi A Nair A Parashar D Ku Z Xiong W Deng H . Oncostatin M receptor-targeted antibodies suppress STAT3 signaling and inhibit ovarian cancer growth. Cancer Res. (2021) 81:5336–52. doi: 10.1158/0008-5472.CAN-21-0483, PMID: 34380633 PMC8530981

[73] Avtanski D Garcia A Caraballo B Thangeswaran P Marin S Bianco J . Resistin induces breast cancer cells epithelial to mesenchymal transition (EMT) and stemness through both adenylyl cyclase-associated protein 1 (CAP1)-dependent and CAP1-independent mechanisms. Cytokine. (2019) 120:155–64. doi: 10.1016/j.cyto.2019.04.016, PMID: 31085453

[74] Chou C-W Huang Y-K Kuo T-T Liu J-P Sher Y-P . An overview of ADAM9: structure, activation, and regulation in human diseases. Int J Mol Sci. (2020) 21:7790. doi: 10.3390/ijms21207790, PMID: 33096780 PMC7590139

[75] Peduto L . ADAM9 as a potential target molecule in cancer. Curr Pharm Des. (2009) 15:2282–7. doi: 10.2174/138161209788682415, PMID: 19601830

[76] Oria VO Lopatta P Schilling O . The pleiotropic roles of ADAM9 in the biology of solid tumors. Cell Mol Life Sci. (2018) 75:2291–301. doi: 10.1007/s00018-018-2796-x, PMID: 29550974 PMC11105608

[77] Huang Y-K Cheng W-C Kuo T-T Yang J-C Wu Y-C Wu H-H . Inhibition of ADAM9 promotes the selective degradation of KRAS and sensitizes pancreatic cancers to chemotherapy. Nat Cancer. (2024) 5:400–19. doi: 10.1038/s43018-023-00720-x, PMID: 38267627

[78] Scribner JA Hicks SW Sinkevicius KW Yoder NC Diedrich G Brown JG . Preclinical evaluation of IMGC936, a next-generation maytansinoid-based antibody-drug conjugate targeting ADAM9-expressing tumors. Mol Cancer Ther. (2022) 21:1047–59. doi: 10.1158/1535-7163.MCT-21-0915, PMID: 35511740

[79] Tanase C Gheorghisan-Galateanu A-A Popescu ID Mihai S Codrici E Albulescu R . CD36 and CD97 in pancreatic cancer versus other Malignancies. Int J Mol Sci. (2020) 21:5656. doi: 10.3390/ijms21165656, PMID: 32781778 PMC7460590

[80] Jia S Zhou L Shen T Zhou S Ding G Cao L . Down-expression of CD36 in pancreatic adenocarcinoma and its correlation with clinicopathological features and prognosis. J Cancer. (2018) 9:578–83. doi: 10.7150/jca.21046, PMID: 29483963 PMC5820925

[81] Tang R Xu J Wang W Meng Q Shao C Zhang Y . Targeting neoadjuvant chemotherapy-induced metabolic reprogramming in pancreatic cancer promotes anti-tumor immunity and chemo-response. Cell Rep Med. (2023) 4:101234. doi: 10.1016/j.xcrm.2023.101234, PMID: 37852179 PMC10591062

[82] Nwosu ZC Ward MH Sajjakulnukit P Poudel P Ragulan C Kasperek S . Uridine-derived ribose fuels glucose-restricted pancreatic cancer. Nature. (2023) 618:151–8. doi: 10.1038/s41586-023-06073-w, PMID: 37198494 PMC10232363

[83] Chen X Cubillos-Ruiz JR . Endoplasmic reticulum stress signals in the tumour and its microenvironment. Nat Rev Cancer. (2021) 21:71–88. doi: 10.1038/s41568-020-00312-2, PMID: 33214692 PMC7927882

[84] Chen X Shi C He M Xiong S Xia X . Endoplasmic reticulum stress: molecular mechanism and therapeutic targets. Signal Transduct Target Ther. (2023) 8:352. doi: 10.1038/s41392-023-01570-w, PMID: 37709773 PMC10502142

[85] Li G Liao C Chen J Wang Z Zhu S Lai J . Targeting the MCP-GPX4/HMGB1 axis for effectively triggering immunogenic ferroptosis in pancreatic ductal adenocarcinoma. Adv Sci (Weinh). (2024) 11:2308208. doi: 10.1002/advs.202308208, PMID: 38593415 PMC11151063

[86] Liao C-Y Li G Kang F-P Lin C-F Xie C-K Wu Y-D . Necroptosis enhances ‘don’t eat me’ signal and induces macrophage extracellular traps to promote pancreatic cancer liver metastasis. Nat Commun. (2024) 15:6043. doi: 10.1038/s41467-024-50450-6, PMID: 39025845 PMC11258255

[87] Xu P Wang C Xiang W Liang Y Li Y Zhang X . P2RY6 has a critical role in mouse skin carcinogenesis by regulating the YAP and β-catenin signaling pathways. J Invest Dermatol. (2022) 142:2334–2342.e8. doi: 10.1016/j.jid.2022.02.017, PMID: 35304248

[88] Girard M Dagenais Bellefeuille S Eiselt É Brouillette R Placet M Arguin G . The P2Y6 receptor signals through Gαq /Ca2+ /PKCα and Gα13 /ROCK pathways to drive the formation of membrane protrusions and dictate cell migration. J Cell Physiol. (2020) 235:9676–90. doi: 10.1002/jcp.29779, PMID: 32420639

[89] Placet M Arguin G Molle CM Babeu J-P Jones C Carrier JC . The G protein-coupled P2Y_6_ receptor promotes colorectal cancer tumorigenesis by inhibiting apoptosis. Biochim Biophys Acta Mol Basis Dis. (2018) 1864:1539–51. doi: 10.1016/j.bbadis.2018.02.008, PMID: 29454075

