# Supplementary figures and images for: Efferocytosis-associated genes serve as prognostic biomarkers for pancreatic ductal adenocarcinoma and identify P2RY6 as a therapeutic target
Source: Front Immunol. 2025 Nov 26;16:1708441. doi: 10.3389/fimmu.2025.1708441 (PMC12689574; doi:10.3389/fimmu.2025.1708441)

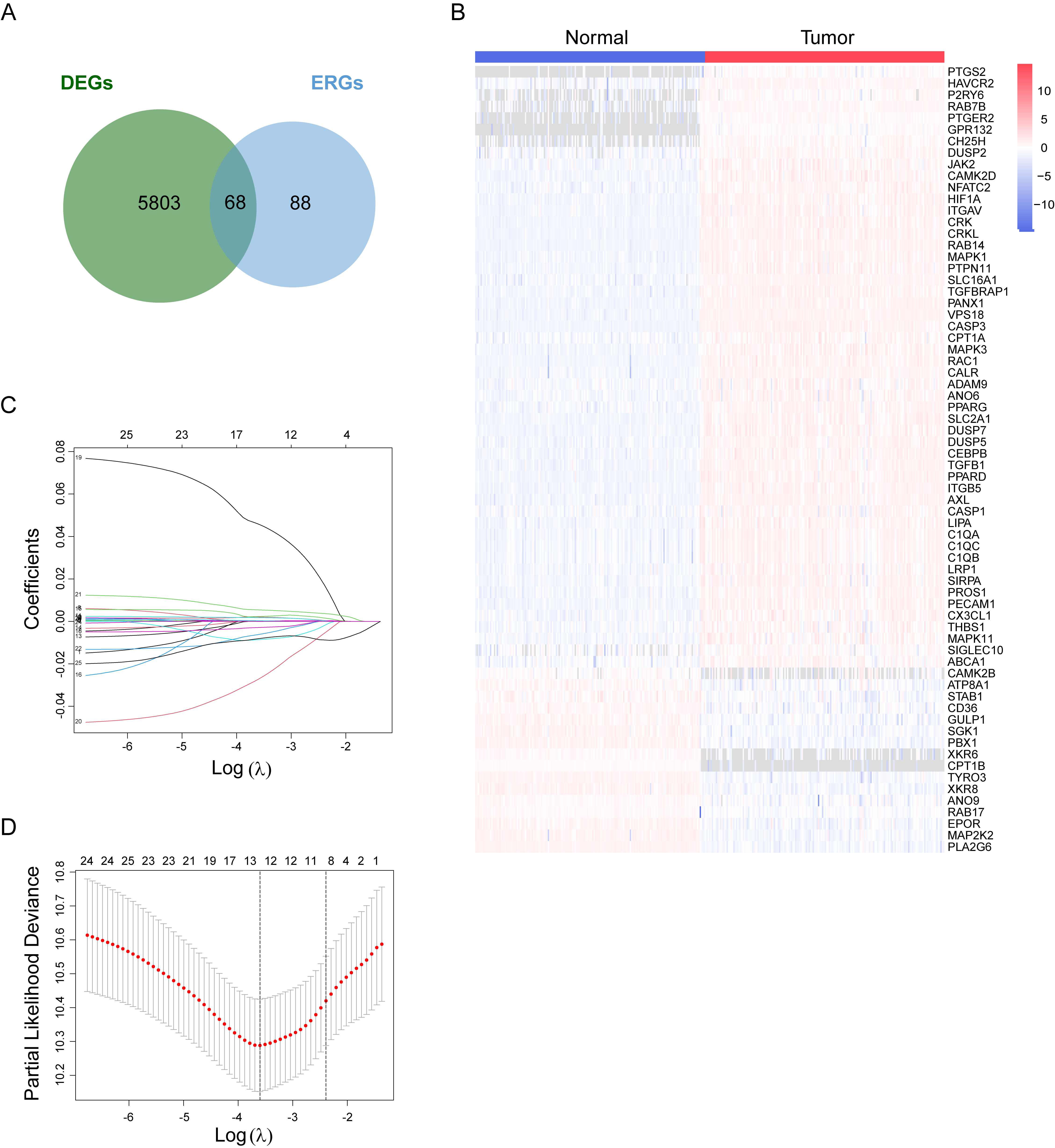

Supplement: Supplementary Figure 1 — Heatmap of DE-ERGs and Optimization of the Prognostic Model. (A) Venn diagram depicts the intersection of the DEGs between tumor and normal tissue (n = 5871) and the efferocytosis-related gene set (n = 158). (B) Heatmap plot shows the expression of DE-ERGs in normal tissue (GTEx dataset, blue) and PDAC tissue (TCGA-PAAD dataset, red). (C) Adjustment path plot from the LASSO regression model, identifies the optimal λ value through ten-fold cross-validation. (D) Results of the LASSO-Cox analysis for the twelve most prognostically significant DE-ERGs. [file Image1.tif]

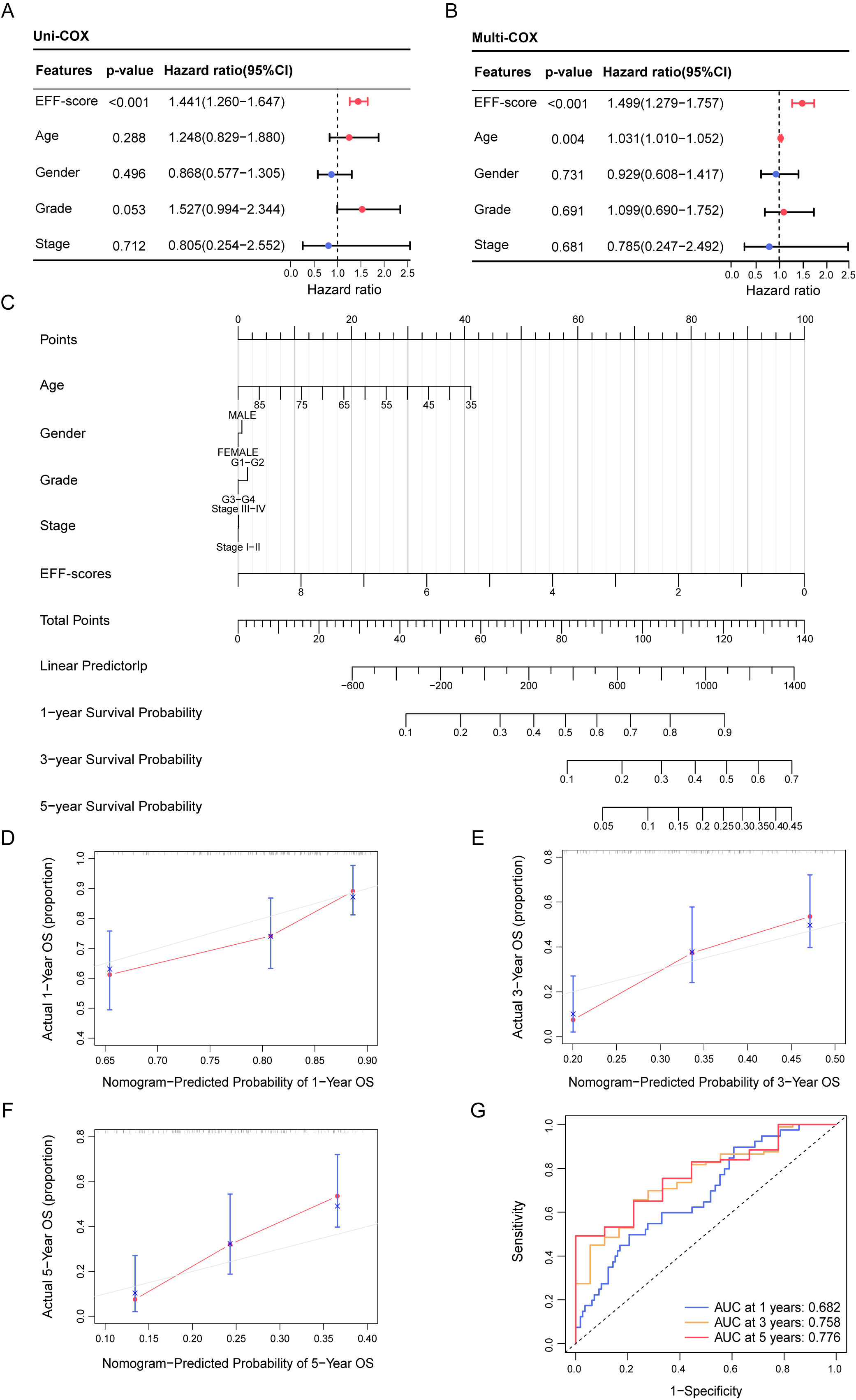

Supplement: Supplementary Figure 2 — Construction and Evaluation of a Nomogram for Predicting the Survival in PDAC Patients from the TCGA Cohort. (A, B) Forest plots of univariate (A) and multivariate (B) Cox regression analysis of PDAC patients based on EFFscore and clinical characteristics, including age, sex, grade, and stage. (C) The prognostic nomogram model was constructed using EFFscore and clinical indicators (age, sex, grade, and stage) to predict 1-year, 3-year, and 5-year OS in PDAC patients. (D-F) Calibration curves assess the performance of the nomogram in predicting 1-year (D), 3-year (E), and 5-year (F) OS. The gray diagonal line represents the ideal prediction, while the blue line indicates the observed prediction. The closer the blue line is to the gray line, the higher the accuracy of the model in predicting survival probabilities. (G) ROC curve for the nomogram demonstrates its predictive performance. [file Image2.tif]

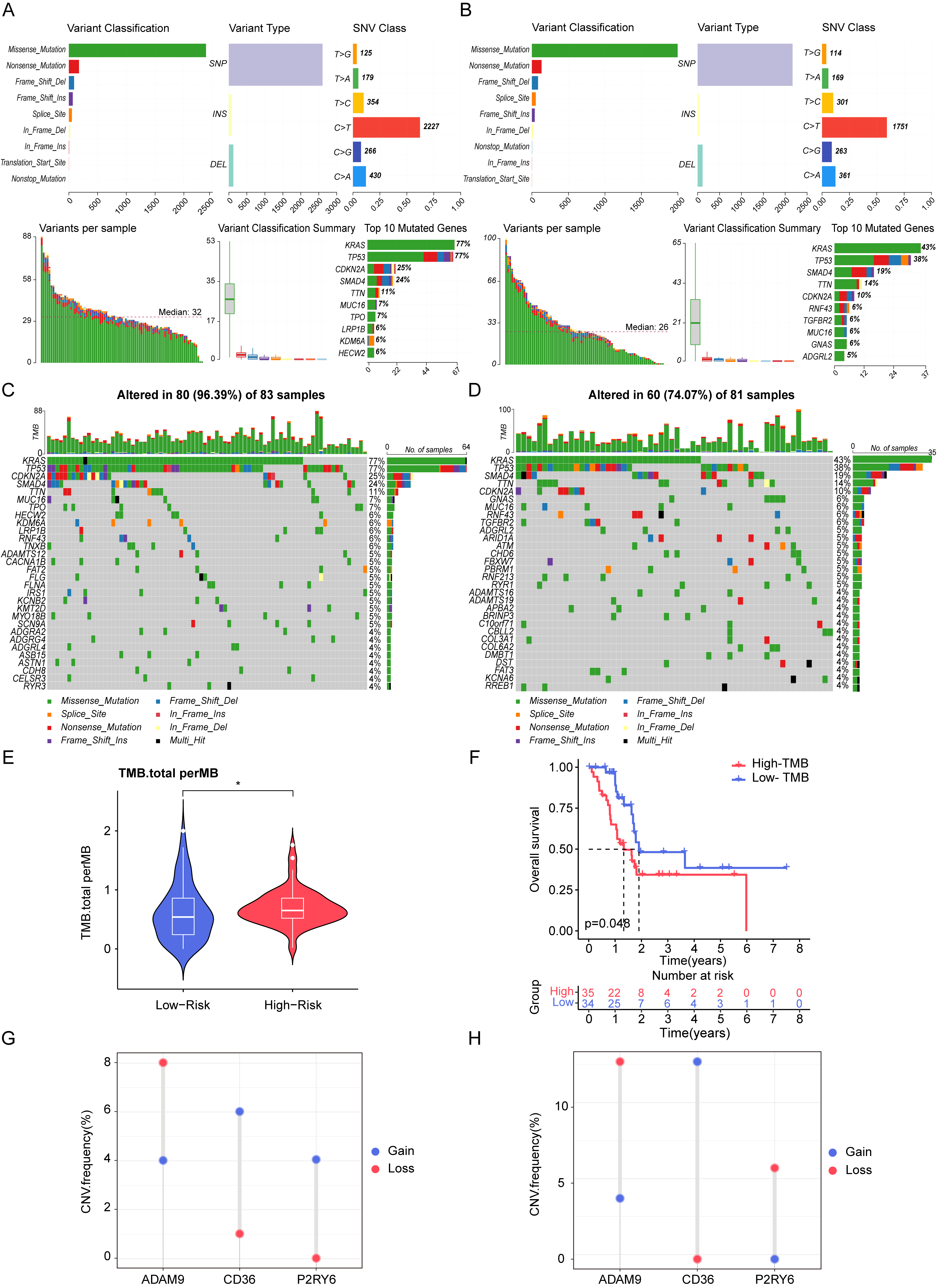

Supplement: Supplementary Figure 3 — Genomic Variation Level in High- and Low-EFFscore Patient Groups. (A, B) Genome mutation profile analysis: Comparative analysis of mutation characteristics in High-EFFscore (A) and Low-EFFscore (B) patients, including mutation classification, mutation type, SNV class, variants per sample, distribution of mutation classifications, and frequently mutated genes. (C, D) Somatic mutation waterfall plot for the High-EFFscore patients (C) and Low-EFFscore patients (D). Each column represents an individual patient, with the top bar plot indicates TMB. Numbers on the right denote the mutation frequency for each gene, and the adjacent bar chart shows the proportion of each variant type. (E) Comparison of total TMB level between High- and Low-EFFscore patients. (F) K-M survival curve shows the survival of PDAC patients with high and low TMB based on the median TMB value. (G–J) CNV frequencies of ADAM9, P2RY6, and CD36 in the High-EFFscore group (G) and Low-EFFscore group (H). Statistical analysis was derived from unpaired t-test, * p < 0.05. [file Image3.tif]

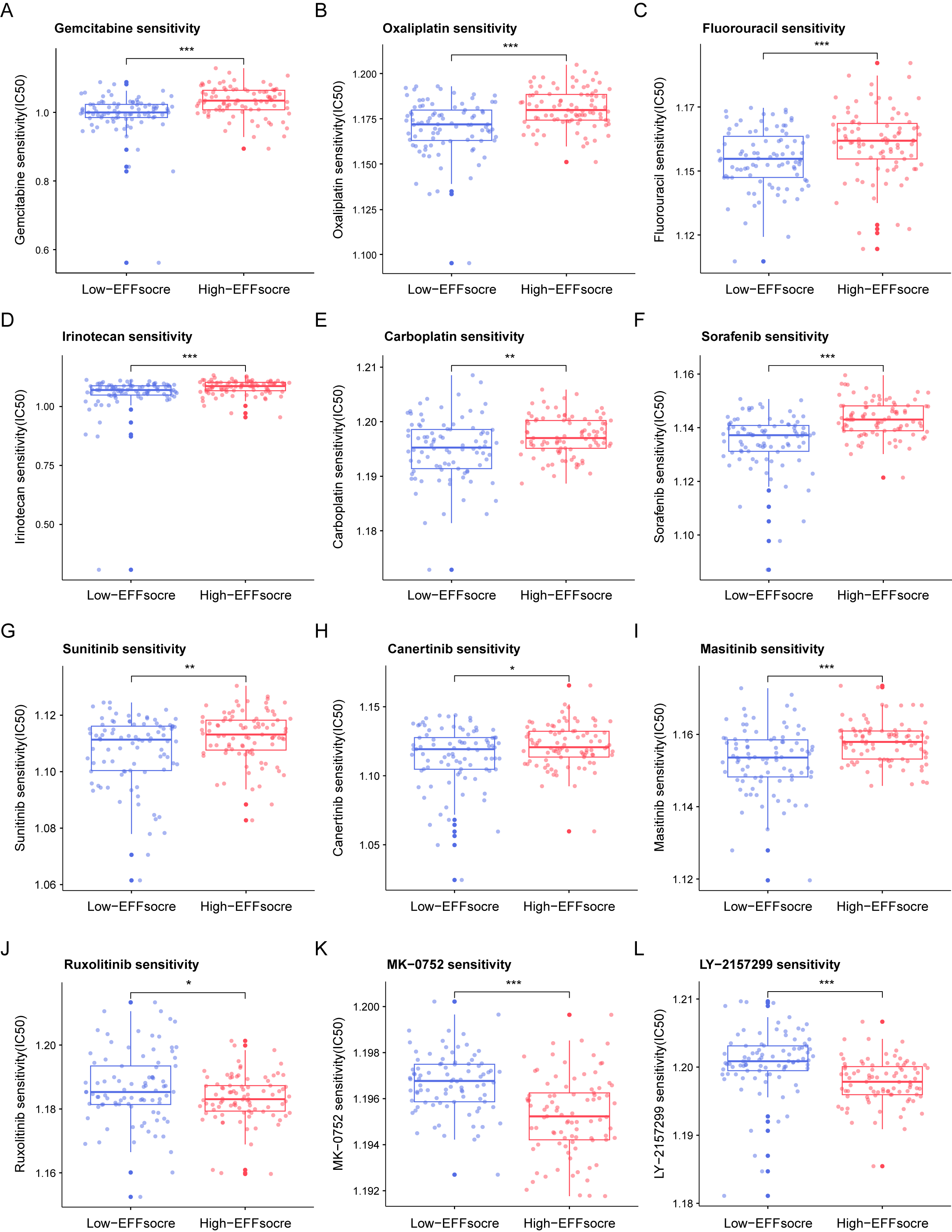

Supplement: Supplementary Figure 4 — Comparison of Drug Sensitivity Between High- and Low-EFFscore Patients. Difference in tumor drug sensitivity between High-and Low-EFFscore patient groups. (A-L) Evaluation of drug sensitivity in High- and Low-EFFscore patients for chemotherapeutic drugs:Gemcitabine (A), Fluorouracil (B), Oxaliplatin (C), Irinotecan (D), Carboplatin (E); tyrosine kinase inhibitors: Sorafenib (F), Sunitinib (G), Canertinib (H), Masitinib (I); JAK inhibitor: Ruxolitinib (J); NOTCH inhibitor: MK-0752 (K); TGF-β inhibitor: LY-2157299 (L). Statistical analyses were derived from Mann-Whitney U test, *p < 0.05, **p < 0.01, ***p < 0.001. [file Image4.tif]

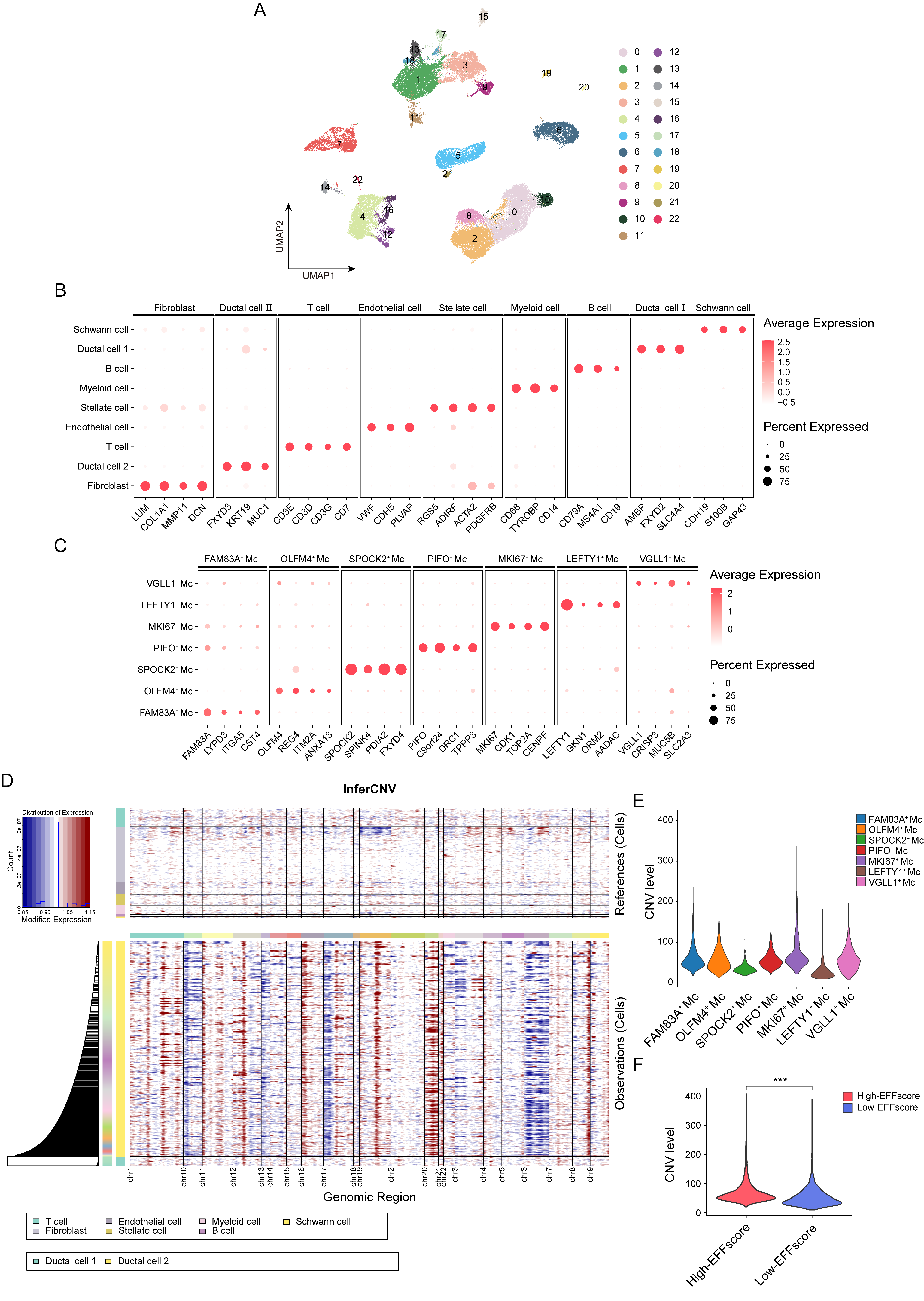

Supplement: Supplementary Figure 5 — Dimensionality Reduction, Clustering and CNV Analysis of Ductal Cells in the GSE194247 Dataset. (A) UMAP plot depicts the clustering of all cells into distinct clusters after dimensionality reduction. (B, C) Bubble plot shows the expression of marker genes across major clusters (B) and PDAC subclusters (C). Bubble size represents the proportion of cells expressing marker genes, while color indicates the average expression of the genes within the cells. (D) CNV analysis of two types of ductal epithelial cell using inferCNV, with all non-ductal cells serving as the reference control. (E) Violin plot shows the CNV level across PDAC subclusters. (F) The difference of CNV between High- and Low-EFFscore subgroup. Statistical analysis was derived from unpaired t-test, ***p < 0.001. [file Image5.tif]

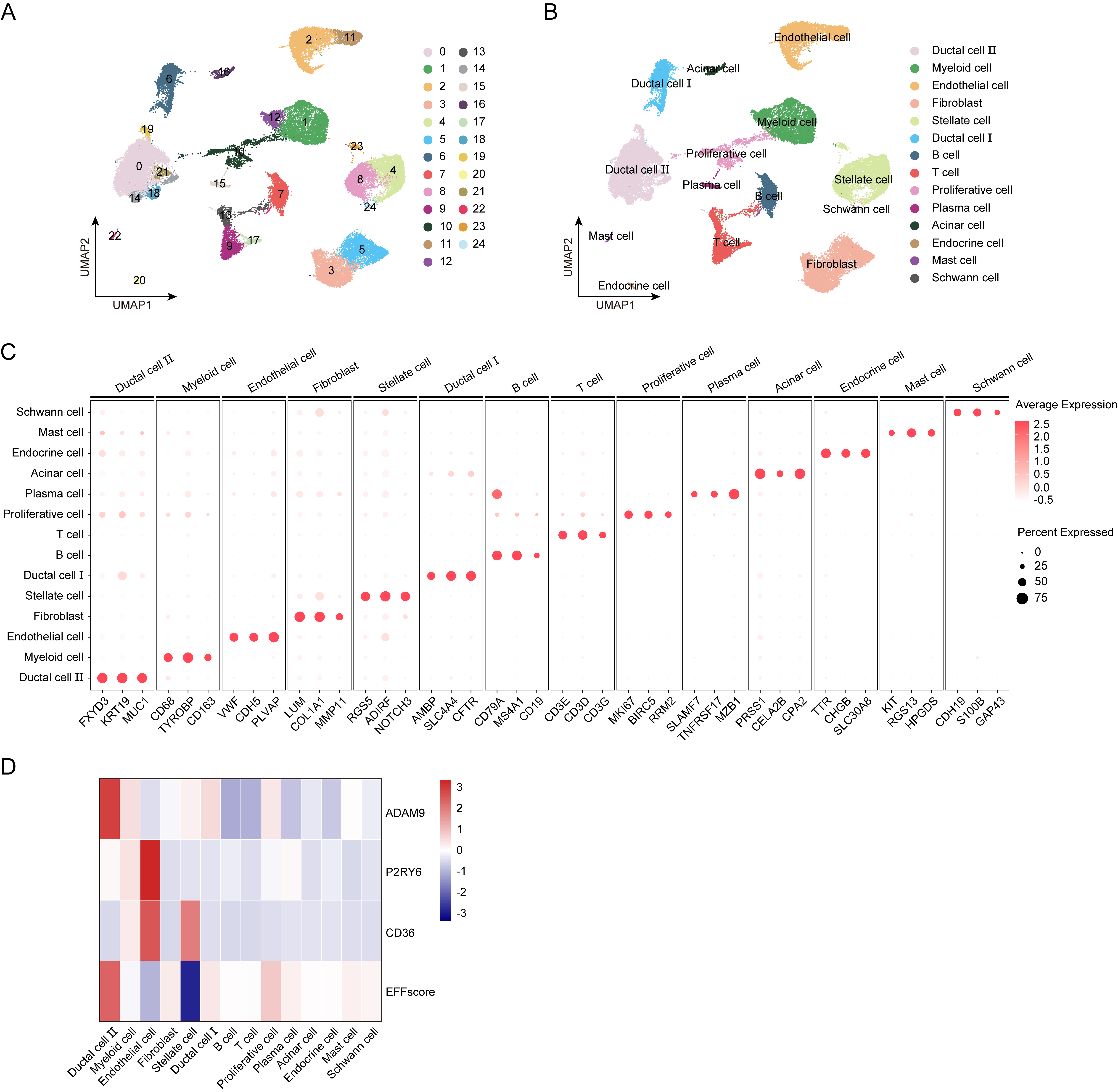

Supplement: Supplementary Figure 6 — Dimensionality Reduction, Clustering and EFFscore Distribution in the CRA001160 Dataset. (A, B) UMAP plots depicts the distribution of cells based on sample origin (A) and the distinct clusters following dimensionality reduction (B). (C) Bubble plot shows the expression profiles of marker genes across clusters. Bubble size represents the proportion of cells expressing marker genes, while color indicates the average expression of the genes within the cells. (D) Heatmap plot shows the Expression and distribution of ADAM9, P2RY6, CD36, and EFFscore across different clusters. [file Image6.tif]

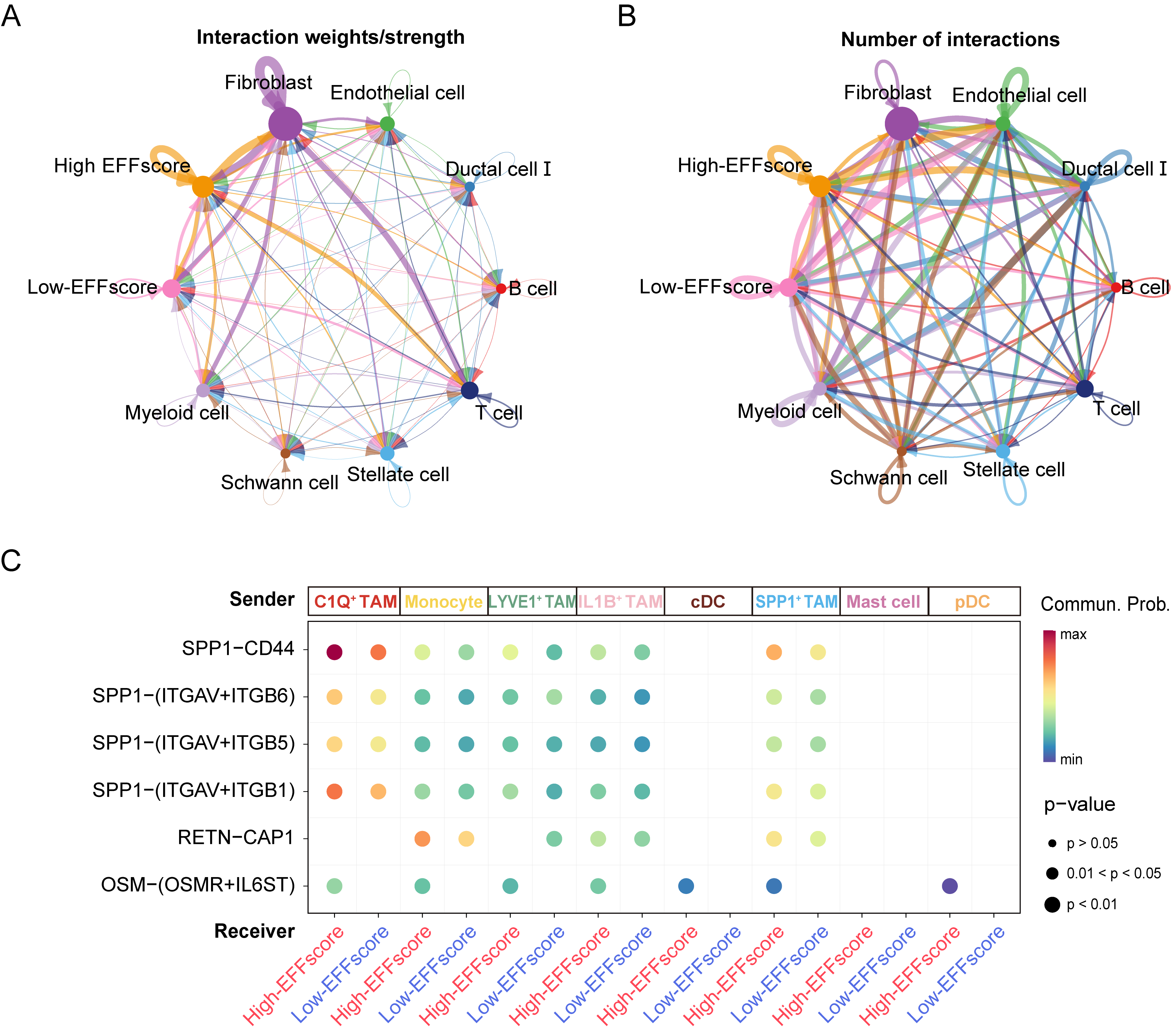

Supplement: Supplementary Figure 7 — Analysis of the Intercellular Communication Network in TME and Ligand-Receptor Interaction Between Myeloid Cell and PDAC cell. (A) CellChat analysis of intercellular communication in TME depicts interaction strength (A) and frequency (B) among various clusters. Node size represents the size of each cluster, while the thickness of the connecting lines indicates the strength and number of interactions. (C) Ligand-receptor interactions between myeloid cells (donors) and distinct EFFscore subgroups (recipient). Dot color represents interaction probability, and dot size indicates the statistical significance (p-value). [file Image7.tif]

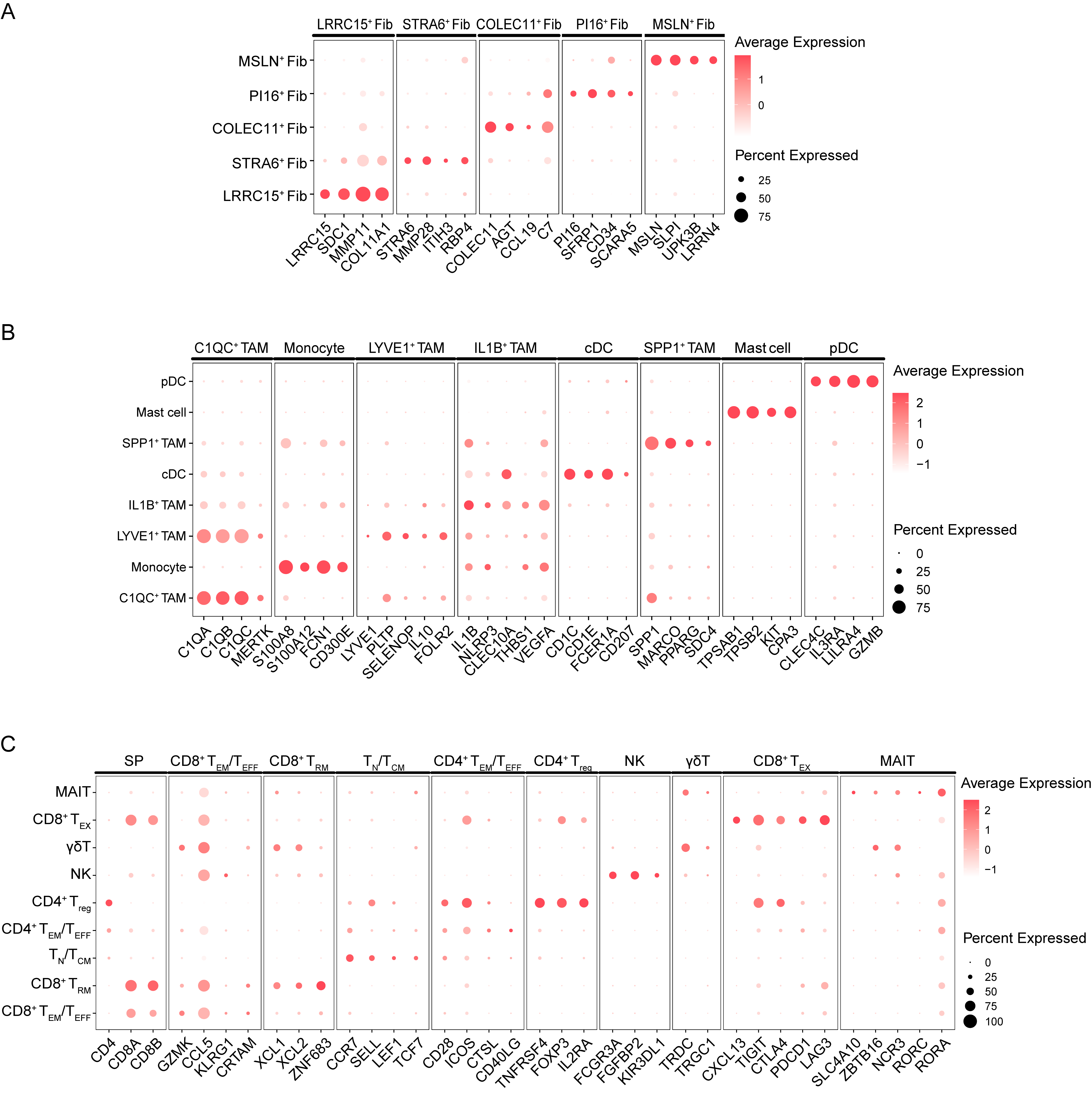

Supplement: Supplementary Figure 8 — Expression of Marker Genes in Fibroblast, Myeloid, and T Cell Subclusters. (A–C) Bubble plot showing the expression patterns of marker genes within fibroblast (A), myeloid cell (B), T cell (C) subclusters. Bubble size represents the proportion of cells expressing marker genes, while color indicates the average expression of the genes within the cells, SP: Single positive. [file Image8.tif]

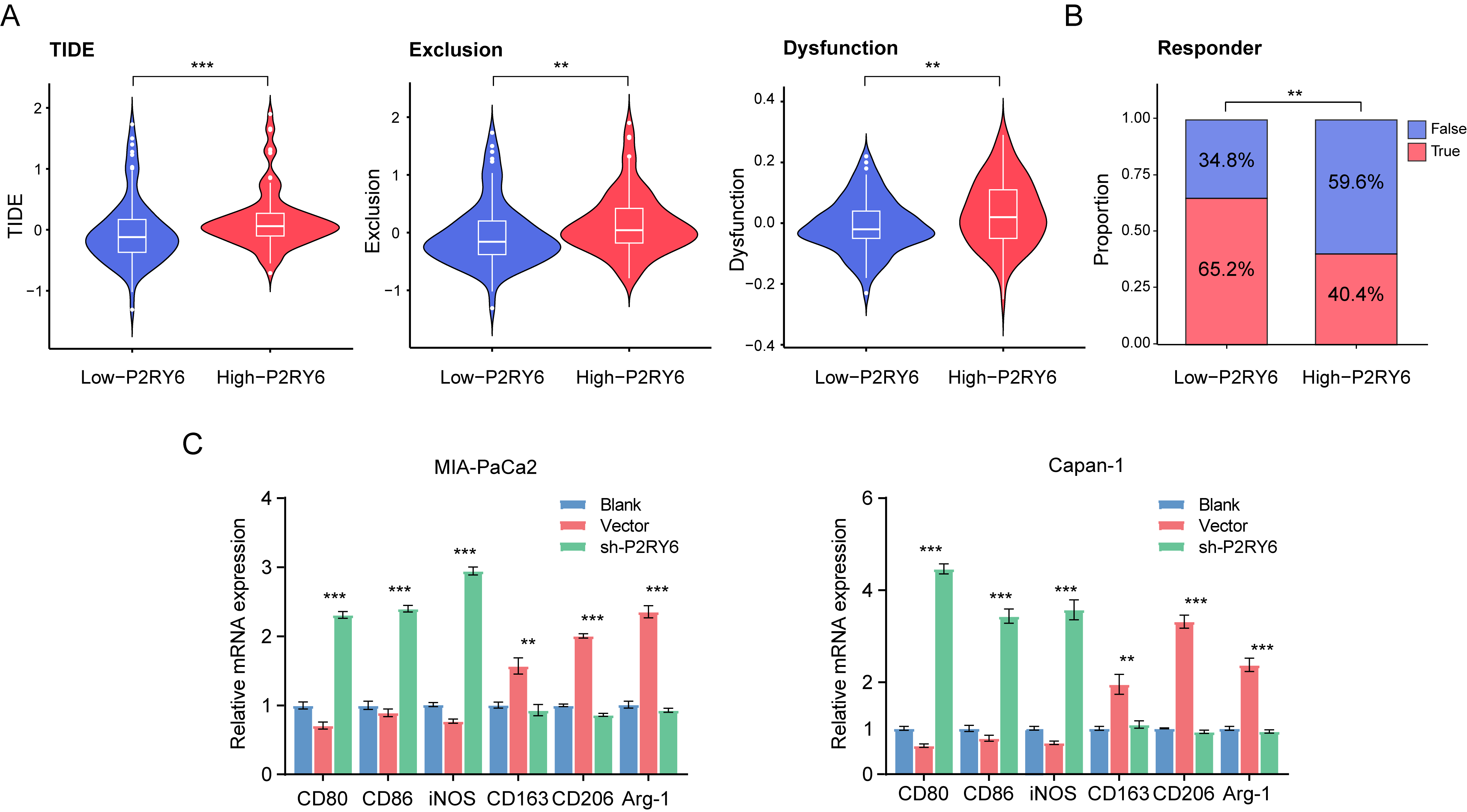

Supplement: Supplementary Figure 9 — P2RY6 Promotes Immune Evasion and Shapes the Immunosuppressive Microenvironment in PDAC. (A, B) TIDE analysis evaluates immunotherapy response, including TIDE score, exclusion score, dysfunction score (A) and immunotherapy response (B), with red indicating responders and blue indicating non-responders. (C) RT-qPCR analysis of markers for M1-like pro-inflammatory and M2-like anti-inflammatory phenotypes after co-culture with MIA-PaCa2 and Capan-1 cells. Statistical analyses include Mann-Whitney U test (A) and Chi-square test (B) and Unpaired t-test (C), error bars represent SD based on three independent experiments (n = 3), ** p < 0.01, *** p < 0.001. [file Image9.tif]
