# Supplementary material for: Efferocytosis-associated genes serve as prognostic biomarkers for pancreatic ductal adenocarcinoma and identify P2RY6 as a therapeutic target
Source: Front Immunol. 2025 Nov 26;16:1708441. doi: 10.3389/fimmu.2025.1708441 (PMC12689574; doi:10.3389/fimmu.2025.1708441)
Supplement: Supplementary file 10 [file Table1.docx]

Table S1. Primer sequences for construct of shRNA vectors and RT-qPCR in the present study

| Genes | Primer sequence (5‘-3’) |  |
| --- | --- | --- |
| sh-P2RY6-1-F | CCGGCCTCTTCTACTTCACCCAGAACTCGAGTTCTGGGTGAAGTAGAAGAGGTTTTTG |  |
| sh-P2RY6-1-R | AATTCAAAAACCTCTTCTACTTCACCCAGAACTCGAGTTCTGGGTGAAGTAGAAGAGG |  |
| sh-P2RY6-2-F | CCGGCGCTTCCTCTTCTATGCCAATCTCGAGATTGGCATAGAAGAGGAAGCGTTTTTG |  |
| sh-P2RY6-2-R | AATTCAAAAACGCTTCCTCTTCTATGCCAATCTCGAGATTGGCATAGAAGAGGAAGCG |  |
| P2RY6-F | CCACAGGCATCCAGCGTAAC |  |
| P2RY6-R | AGGAAGCCGATGACAGTGAGAG |  |
| CD36-F | GGTGCTGTCCTGGCTGTGTT |  |
| CD36-R | TGTTGCTGCTGTTCATCATCACTT |  |
| ADAM9-F | CATCGGGTTCCAGAAACTTTAGCA |  |
| ADAM9-R | GCGTCCACCAACTTATTACCACAG |  |
| HLA-A-F | TGTCTTCCCAGCCCACCATCC |  |
| HLA-A-R | CATCACGGCAGCGACCACAG |  |
| HLA-B-F | CCCAGTCCACCGTCCCCATC |  |
| HLA-B-R | GCTCTTGTCCAGAAGGCACCAC |  |
| HLA-C-F | TCTACCCTGCGGAGATCACACTG |  |
| HLA-C-R | GCTCTTGTCCAGAAGGCACCAC |  |
| HLA-DRA-F | CTGGCGGCTTGAAGAATTTGGA |  |
| HLA-DRA-R | GGAGGTACATTGGTGATCGGAGTA |  |
| HLA-DRB1-F | CAGCGGCGAGTCCAACCTAA |  |
| HLA-DRB1-R | AACCACTCACAGAGCAGACCAG |  |
| HLA-DQA1-F | GGACCTGGAGAGGAAGGAGACT |  |
| HLA-DQA1-R | TCATTGGTAGCAGCGGTAGAGTT |  |
| HLA-DQB1-F | CTTGATGCTGGCGATGCTGAG |  |
| HLA-DQB1-R | TCCGTCCCGTTGGTGAAGTAG |  |
| HLA-DPA1-F | CGTCTGGCATCTGGAGGAGTT |  |
| HLA-DPA1-R | AGTGTGGTTGGAACGCTGGAT |  |
| HLA-DPB1-F | TGTCCACCAACCTGATCCGTAAT |  |
| HLA-DPB1-R  DDIT3-F | CAGACTGTGCCTTCCACTCCA  CTTCTCTGGCTTGGCTGACTGA |  |
| DDIT3-R | TTGGTCTTCCTCCTCTTCCTCCT |  |
| HSPA5-F | TGCGTCGGCGTGTTCAAGA |  |
| HSPA5-R | GGTTGGAGGTGAGCTGGTTCT |  |
| PERK-F | TTCTCCTCCAAGACCAACCACTT |  |
| PERK-R | GGTACATCGTCCATTCATCCAGTC |  |
| ERO1A-F | ACTGTGCTGTCAAACCATGTCAAT |  |
| ERO1A-R | ATCCACTGCTCCAAGTCGTTCA |  |
| PPP1R15A-F | ACCTCTACTTCTGCCTTGTCTCC |  |
| PPP1R15A-R | ACGCCTCTCCTGAACGATACTC |  |
| Genes | Primer sequence (5‘-3’) |  |
| ATF4-F | CCTTCACCTTCTTACAACCTCTTC |  |
| ATF4-R | TAGTCTGGCTTCCTATCTCCTTCA |  |
| ATF6-F | GGCAGGACTACGAAGTGATGATG |  |
| ATF6-R | GAGGTAAGGAGGAACTGACGAACT |  |
| IRE1α-F | GGAGGGTCTGAGGAAGGTGATG |  |
| IRE1α-R | GGAGAGGCATAGAGGCTGGTAG |  |
| EIF2α-F | TGAGGTGGAAGATGTAGTGATGGT |  |
| EIF2α-R | ACGGATACGCCTTCTGGATAATTC |  |
| XBP1-F | ATGGATTCTGGCGGTATTGACTCT |  |
| XBP1-R | GAAAGGGAGGCTGGTAAGGAACT |  |
| TNFRSF10B-F | CGATGCTGATAAAGTGGGTCAACA |  |
| TNFRSF10B-R | GCTCAACAAGTGGTCCTCAATCTT |  |
| TRIB3-F | GTCTGGTCCTGCGTGATCTCAA |  |
| TRIB3-R | GCTGCCTTGCCCGAGTATGA |  |
| BCL2L11-F | AGAGTTGCGGCGTATTGGAGA |  |
| BCL2L11-R | TGGTCTTCGGCTGCTTGGTAA |  |
| BBC3-F | TACGAGCGGCGGAGACAAGA |  |
| BBC3-R | AGGGCAGGAGTCCCATGATGA |  |
| 18S-F | TTCGAACGTCTGCCCTATCAA |  |
| 18S-R | ATGGTAGGCACGGCGACTA |  |
