# Supplementary material for: Efferocytosis-associated genes serve as prognostic biomarkers for pancreatic ductal adenocarcinoma and identify P2RY6 as a therapeutic target
Source: Front Immunol. 2025 Nov 26;16:1708441. doi: 10.3389/fimmu.2025.1708441 (PMC12689574; doi:10.3389/fimmu.2025.1708441)
Supplement: Supplementary file 11 [file Table2.docx]

Table S2. The results of univariate Cox regression analysis based on differentially expressed efferocytosis-related genes.

| Genes | HR | 95% CI of HR | p value |
| --- | --- | --- | --- |
| CPT1B | 0.5900 | 0.3049-1.1417 | 0.1172 |
| RAB14 | 1.0060 | 0.9993-1.0127 | 0.0801 |
| VPS18 | 0.9868 | 0.9678-1.0062 | 0.1821 |
| CASP3 | 1.0117 | 0.9997-1.0238 | 0.0558 |
| CRKL | 1.0031 | 0.9932-1.0130 | 0.5413 |
| PLA2G6 | 0.9666 | 0.9228-1.0124 | 0.1505 |
| PANX1 | 1.0221 | 1.0086-1.0358 | 0.0013 |
| SLC16A1 | 1.0145 | 1.0063-1.0227 | 0.0005 |
| CRK | 1.0082 | 0.9977-1.0188 | 0.1265 |
| RAC1 | 1.0016 | 1.0004-1.0028 | 0.0105 |
| AXL | 1.0053 | 1.0017-1.0088 | 0.0035 |
| PTGER2 | 1.0034 | 0.9898-1.0172 | 0.6255 |
| GPR132 | 1.0181 | 0.9874-1.0497 | 0.2510 |
| TGFBRAP1 | 1.0139 | 0.9814-1.0475 | 0.4069 |
| CALR | 1.0009 | 1.0000-1.0018 | 0.0397 |
| ITGB5 | 1.0029 | 1.0014-1.0044 | 0.0001 |
| PPARD | 1.0082 | 1.0009-1.0155 | 0.0275 |
| TYRO3 | 1.0720 | 0.9853-1.1664 | 0.1063 |
| RAB7B | 1.0028 | 0.9851-1.0208 | 0.7593 |
| MAPK1 | 1.0100 | 1.0019-1.0182 | 0.0159 |
| DUSP5 | 1.0024 | 1.0001-1.0046 | 0.0365 |
| SLC2A1 | 1.0011 | 1.0000-1.0021 | 0.0531 |
| TGFB1 | 1.0008 | 0.9962-1.0054 | 0.7345 |
| DUSP7 | 1.0193 | 1.0048-1.0341 | 0.0092 |
| PTGS2 | 1.0039 | 1.0011-1.0068 | 0.0073 |
| MAPK3 | 1.0031 | 0.9983-1.0078 | 0.2039 |
| CAMK2D | 1.0055 | 0.9890-1.0222 | 0.5170 |
| PROS1 | 1.0029 | 0.9915-1.0143 | 0.6222 |
| PBX1 | 1.0105 | 0.9762-1.0459 | 0.5529 |
| HIF1A | 1.0035 | 1.0007-1.0064 | 0.0144 |
| ITGAV | 1.0054 | 1.0021-1.0088 | 0.0015 |
| HAVCR2 | 1.0062 | 0.9936-1.0189 | 0.3379 |
| PTPN11 | 1.0039 | 0.9955-1.0124 | 0.3656 |
| XKR6 | 1.0380 | 0.7883-1.3667 | 0.7904 |
| PECAM1 | 0.9998 | 0.9958-1.0038 | 0.9140 |
| CEBPB | 1.0012 | 0.9998-1.0026 | 0.0950 |
| SGK1 | 1.0050 | 0.9967-1.0134 | 0.2374 |
| C1QA | 1.0003 | 0.9997-1.0008 | 0.3188 |
| PPARG | 1.0056 | 0.9995-1.0117 | 0.0708 |
| XKR8 | 0.9484 | 0.8624-1.0430 | 0.2750 |
| MAP2K2 | 0.9915 | 0.9833-0.9998 | 0.0441 |
| NFATC2 | 0.9922 | 0.9743-1.0104 | 0.3987 |
| LIPA | 1.0003 | 0.9965-1.0041 | 0.8749 |
| C1QC | 1.0004 | 0.9997-1.0010 | 0.2764 |
| ANO6 | 1.0160 | 1.0074-1.0247 | 0.0002 |
| CH25H | 0.9898 | 0.9731-1.0067 | 0.2356 |
| SIRPA | 1.0001 | 0.9917-1.0086 | 0.9783 |
| GULP1 | 1.0448 | 1.0062-1.0849 | 0.0224^*^ |
| EPOR | 0.9621 | 0.9356-0.9894 | 0.0067 |
| C1QB | 1.0003 | 0.9998-1.0008 | 0.2399 |
| LRP1 | 1.0021 | 0.9991-1.0051 | 0.1750 |
| JAK2 | 1.0067 | 0.9747-1.0396 | 0.6863 |
| CPT1A | 1.0062 | 0.9989-1.0136 | 0.0949 |
| CX3CL1 | 1.0027 | 0.9986-1.0069 | 0.1993 |
| DUSP2 | 1.0042 | 0.9950-1.0136 | 0.3690 |
| RAB17 | 0.9769 | 0.9616-0.9924 | 0.0037^*^ |
| P2RY6 | 1.0538 | 1.0202-1.0885 | 0.0015 |
| CD36 | 0.9726 | 0.9481-0.9976 | 0.0323 |
| ADAM9 | 1.0121 | 1.0061-1.0180 | 0.0001 |
| MAPK11 | 0.9761 | 0.9578-0.9947 | 0.0122 |
| CASP1 | 1.0148 | 0.9977-1.0322 | 0.0905 |
| ABCA1 | 1.0064 | 0.9833-1.0301 | 0.5910 |
| STAB1 | 0.9938 | 0.9798-1.0080 | 0.3918 |
| THBS1 | 1.0008 | 1.0001-1.0015 | 0.0289 |
| ANO9 | 0.9975 | 0.9840-1.0113 | 0.7253 |
| ATP8A1 | 0.9685 | 0.9395-0.9984 | 0.0388 |
| SIGLEC10 | 0.9992 | 0.9738-1.0252 | 0.9503 |
| CAMK2B | 0.9540 | 0.9187-0.9907 | 0.0144 |
