# Supplementary material for: Efferocytosis-associated genes serve as prognostic biomarkers for pancreatic ductal adenocarcinoma and identify P2RY6 as a therapeutic target
Source: Front Immunol. 2025 Nov 26;16:1708441. doi: 10.3389/fimmu.2025.1708441 (PMC12689574; doi:10.3389/fimmu.2025.1708441)
Supplement: Supplementary file 12 [file Table3.docx]

Table S3. Correlation between P2RY6 expression and xCell-estimated cell fractions in PDAC samples.

| Cell type (xCell) | Correlation | P value |
| --- | --- | --- |
| aDC | 0.3483 | 1.89E-06 |
| Monocytes | 0.3426 | 2.86E-06 |
| Macrophages M1 | 0.3303 | 6.72E-06 |
| DC | 0.3275 | 8.13E-06 |
| Tgd cells | 0.3099 | 2.56E-05 |
| Macrophages | 0.3004 | 4.61E-05 |
| cDC | 0.2760 | 0.0002 |
| pro B-cells | 0.2668 | 0.0003 |
| Th2 cells | 0.2612 | 0.0004 |
| mv Endothelial cells | 0.2601 | 0.0005 |
| Endothelial cells | 0.2516 | 0.0007 |
| ly Endothelial cells | 0.2496 | 0.0008 |
| iDC | 0.2491 | 0.0008 |
| CD4^+^ naive T-cells | 0.2394 | 0.0013 |
| CD4^+^ memory T-cells | 0.2378 | 0.0014 |
| Fibroblasts | 0.2376 | 0.0014 |
| B-cells | 0.2371 | 0.0014 |
| Tregs | 0.2289 | 0.0021 |
| naive B-cells | 0.2251 | 0.0025 |
| CD8^+^ Tcm | 0.2142 | 0.0041 |
| Memory B-cells | 0.2128 | 0.0043 |
| Macrophages M2 | 0.2106 | 0.0048 |
| Epithelial cells | 0.2051 | 0.0387 |
| CD8+ Tem | 0.1917 | 0.0103 |
| Class-switched memory B-cells | 0.1856 | 0.0131 |
| CD4^+^ Tem | 0.1552 | 0.0386 |
| CD8^+^ T-cells | 0.1544 | 0.0396 |
| CD4^+^ T-cells | 0.1490 | 0.0471 |
| pDC | 0.1385 | 0.0653 |
| Mast cells | 0.1330 | 0.0768 |
| CD8^+^ naive T-cells | 0.1115 | 0.1386 |
| Pericytes | 0.1100 | 0.1437 |
| Basophils | 0.0957 | 0.2038 |
| CD4^+^ Tcm | 0.0912 | 0.2258 |
| Eosinophils | 0.0897 | 0.2338 |
| Neutrophils | 0.0843 | 0.2635 |
| NKT | 0.0829 | 0.2712 |
| NK cells | 0.0783 | 0.2990 |
| Th1 cells | 0.0577 | 0.4440 |
| Plasma cells | -0.0309 | 0.6824 |
| Neurons | -0.1404 | 0.0615 |
